# Optimum tacrolimus trough levels for enhanced graft survival and safety in kidney transplantation: a retrospective multicenter real-world evidence study

**DOI:** 10.1097/JS9.0000000000001800

**Published:** 2024-06-17

**Authors:** Ahram Han, Ae Jeong Jo, Hyunwook Kwon, Young Hoon Kim, Juhan Lee, Kyu Ha Huh, Kyo Won Lee, Jae Berm Park, Eunju Jang, Sun Cheol Park, Joongyub Lee, Jeongyun Lee, Younghye Kim, Mohamed Soliman, Sangil Min

**Affiliations:** a Division of Transplantation and Vascular Surgery, Department of Surgery, Seoul National University Hospital, Seoul; b Department of Information Statistics, Andong National University, Andong; c Department of Kidney and Pancreases Transplantation, Department of Surgery, Asan Medical Center, University of Ulsan College of Medicine, Seoul; d Department of Surgery, Shinchon Severance Hospital, Yonsei University College of Medicine, Seoul; e Department of Surgery, Samsung Medical Center, Sungkyunkwan University School of Medicine, Seoul; f Division of Vascular and Transplant Surgery, Department of Surgery, College of Medicine, The Catholic University of Korea, Seoul; g Department of Preventive Medicine, Seoul National University College of Medicine, Seoul; h Medical Affairs Department, Astellas Pharma Korea, Seoul; i Medical Affairs Department, Astellas Pharma Singapore Pte Ltd., Singapore

**Keywords:** graft rejection, kidney transplantation, safety, tacrolimus

## Abstract

**Background:**

The current study aimed to determine the optimal tacrolimus trough levels for balancing graft survival and patient safety following kidney transplantation.

**Materials and methods:**

We conducted a retrospective cohort study involving 11 868 kidney transplant recipients from five medical centers. The association between tacrolimus exposures (periodic mean trough level, coefficient of variability, time in therapeutic range) and composite allograft outcome (de novo donor-specific antibody, biopsy-proven rejection, kidney dysfunction, and graft failure), as well as safety outcomes (severe infection, cardiovascular events, malignancy, and mortality) were assessed. Data were sourced from Clinical Data Warehouses and analyzed using advanced statistical methods, including Cox marginal structural models with inverse probability treatment weighting.

**Results:**

Tacrolimus levels of 5.0–7.9 ng/ml and 5.0–6.9 ng/ml during the 2–12 month and 12–72 month post-transplantation periods, respectively, were associated with reduced risks of composite allograft outcomes. During the first post-transplant year, the adjusted hazard ratios (aHR) for composite allograft outcomes were 0.69 (95% CI 0.55–0.85, *P*<0.001) for 5.0–5.9 ng/ml; 0.81 (95% CI 0.67–0.98, *P*=0.033) for 6.0–6.9 ng/ml; and 0.73 (95% CI 0.60–0.89, *P*=0.002) for 7.0–7.9 ng/ml (compared to levels ≥8.0 ng/ml). For the 6-year composite outcomes, aHRs were 0.68 (95% CI 0.53–0.87, *P*=0.002) for 5.0–5.9 ng/ml and 0.65 (95% CI 0.50–0.85, *P*=0.001) for 6.0–6.9 ng/ml. These optimal ranges showed reduced rates of severe infection (6 years), malignancy (6 years), and mortality (1 year).

**Conclusion:**

This multicenter study provides robust evidence for optimal tacrolimus trough levels during the periods 2–12 and 12–72 months following kidney transplantation.

## Graphical abstract

**Figure:**
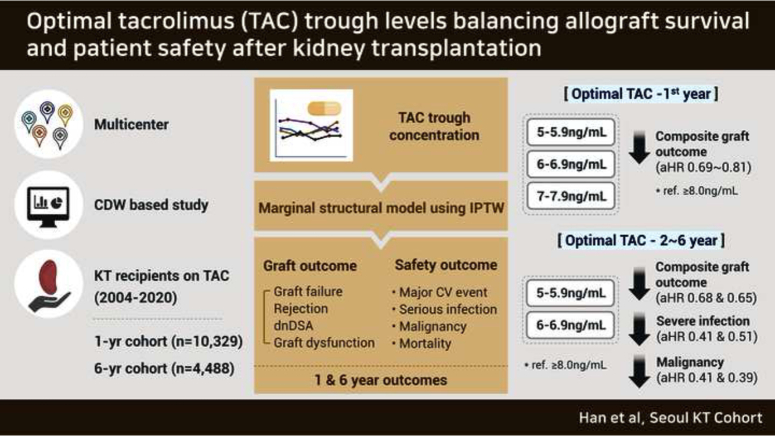

## Introduction

Highlights

- A multicenter study employing institutional Clinical Data Warehouses (CDW) to ascertain optimal tacrolimus trough levels that balance efficacy and safety in kidney transplant recipients.
- Identified optimal tacrolimus trough levels of 5.0–7.9 ng/ml during the 2–12 month post-transplant period and 5.0–6.9 ng/ml during the 12–72 month period, correlating with improved graft outcomes and reduced risks of safety outcomes, including infections, cardiovascular events, malignancies, and mortality.
- Emphasizes the clinical significance of maintaining tacrolimus within these specified ranges, proposing an adjustment of immunosuppressive protocols in kidney transplantation to optimize graft longevity and minimize treatment-related adverse effects.

Kidney transplantation remains the gold standard for managing end-stage renal disease, offering superior patient survival and quality of life^1,2^. Advances in immunosuppressive regimens have led to decreased rates of acute rejection and better short-term outcomes^3^. Currently, the most common regimen combines tacrolimus with mycophenolate derivatives and steroids^4,5^.

The principal challenge in immunosuppression is achieving a balance between under-immunosuppression and over-immunosuppression; under-immunosuppression can lead to graft rejection, while over-immunosuppression can lead to off-target toxicities and infection^6^. Tacrolimus, the cornerstone of modern immunosuppressive regimens^4^, has a narrow therapeutic index and is associated with a range of adverse effects, including kidney dysfunction, hypertension, and dyslipidemia^7–9^. Though post-transplant tacrolimus trough levels are regularly monitored, optimal levels are not well defined^10^.

Historically, tacrolimus trough level targets were proposed to range from 5 to 20 ng/ml^11–13^. The Efficacy Limiting Toxicity Elimination (ELITE)-Symphony trial later advocated that targeting lower levels of 5–10 ng/ml tacrolimus led to superior effectiveness with acceptable adverse effects compared to previous cyclosporine-based or sirolimus-based regimens^3,14^. Following the Symphony trial, further studies, mostly of limited size and retrospective in nature, have produced conflicting results concerning tacrolimus trough levels and graft outcomes^15–21^. Larger registry studies, based on fragmented trough-level data^22,23^, have failed to capture the dynamic fluctuations of tacrolimus over time. More recently, concepts of intrapatient variability (IPV) and time in therapeutic range (TTR) have been introduced to address the varying nature of tacrolimus trough levels^24,25^; however, target ranges of IPV and TTR are not well defined. Furthermore, data on optimal tacrolimus levels beyond the initial post-transplant year are remarkably scarce, highlighting a substantial gap in knowledge within the field.

The task of optimizing tacrolimus dosing in immunosuppression therapy extends beyond mere monitoring of drug levels; it requires holistic consideration of a multitude of clinical factors, including patient age, cardiovascular health, infection status, renal function, recent graft rejections, and donor-specific antibodies. These factors are pivotal not only in immediate clinical decision-making regarding tacrolimus dosing but also significantly impact both transplant and patient outcomes. The dynamic and multifactorial nature of these considerations emphasizes the need for an analytical approach capable of elucidating the complex relationships among these covariates, tacrolimus exposure, and transplant outcomes.

In response to this complexity, our study advocates for the utilization of Clinical Data Warehouses (CDW) as a pivotal resource. CDWs provide an advanced platform for big data analytics, facilitating the comprehensive analysis of longitudinal observational data that mirrors real-world clinical practice^26,27^. The CDWs from the participating five medical centers, upon which our database is based, are automatically updated with a one-day time lag, capturing a vast array of electronic health record (EHR) data, including text medical records, anesthesia records, nursing notes, laboratory data, pathology reports, and unstructured imaging/diagnostic test interpretations. We utilized the CDWs to systematically collect data on 483 variables across our study cohort, thereby minimizing human error and reducing bias during data collection. The longitudinal data were comprehensive, covering from 1 year pre-surgery to 6 years post-transplant or until the end of each patient’s follow-up period.

In addition, to adequately model the intricate and time-varying interplay between covariates, tacrolimus exposure, and transplantation outcomes, we employed advanced statistical methodologies, specifically Cox Marginal Structural Models (MSM) that utilize stabilized weights calculated via the Inverse Probability of Treatment Weighting (IPTW) method^28,29^. This approach allows for adjustments of time-dependent confounders, thereby mitigating potential biases in estimating the impact of tacrolimus exposure on graft and patient outcomes.

By integrating comprehensive CDW data with sophisticated statistical analysis, we sought to elucidate the optimal tacrolimus levels that would balance graft survival with patient safety. By considering the complex clinical decision-making framework and the multifactorial influences on immunosuppression outcomes, our research aims to provide actionable insights for the refinement of tacrolimus management protocols in kidney transplantation.

## Materials and methods

This retrospective multicenter cohort study (ClinicalTrials.gov, number NCT06348446) used CDW data to investigate the relationship between tacrolimus exposure and both short-term and long-term graft and patient outcomes in kidney transplant recipients. The study protocol was approved by the institutional review boards of the five participating centers in Korea.

### Study population

The study cohort included patients who underwent kidney-only transplants between January 2005 and December 2020. Patients were included if they were being administered oral tacrolimus at the start of the defined cohort time intervals, specifically 2 months post-transplant for the 1-year outcomes and 12 months post-transplant for the 6-year outcomes. We excluded patients who experienced graft failure or death before these time intervals and those who received other solid organ transplants during the study period. In patients who received multiple kidney transplants during the study period, only the first transplant was included.

### Outcome variables and study endpoint

The primary endpoint was a composite of 1-year allograft outcomes, consisting of biopsy-proven rejection (BPR), kidney dysfunction [estimated glomerular filtration rate (eGFR) <30 ml/min/1.73 m^2^], the development of anti-human leukocyte antigen (HLA) de novo donor-specific antibodies (dnDSA), and death-censored graft failure, occurring 2–12 months post-transplant. Secondary endpoints were composite allograft outcomes at 12–72 months and safety outcomes (severe infection, cardiovascular events, malignancies, and mortality) occurring 2–12 months and 12–72 months post-transplant. Detailed definitions of the outcome variables are provided in the Supplementary Methods, Supplemental Digital Content 1, http://links.lww.com/JS9/C773. Follow-up was censored at 1 or 6 years post-transplant, loss to follow-up, or by 31 December 2021, whichever occurred first.

### Tacrolimus exposure variables

Tacrolimus exposure was assessed through several variables, including the periodic mean of tacrolimus trough level, the coefficient of variability (CV) as a measure of IPV and TTR. The periodic mean was determined from multiple outpatient tacrolimus trough level measurements. If there were more than one measurement on the same day, the lower value was selected. Bi-monthly means within the first year post-transplant (2–12 months) were used for the analysis of its association with early post-transplant outcomes, and annual means during the subsequent 5 years (12–72 months) were used to evaluate 6-year outcomes. The periodic mean of tacrolimus trough levels was categorized into seven groups: <3.0 ng/ml, 3.0–3.9 ng/ml, 4.0–4.9 ng/ml, 5.0–5.9 ng/ml, 6.0–6.9 ng/ml, 7.0–7.9 ng/ml, and ≥8.0 ng/ml.

CV for tacrolimus trough concentrations was calculated as the standard deviation to mean ratio, categorized into quartiles. TTR was assessed using the Rosendaal method^30,31^, with therapeutic tacrolimus levels set at 7.0–10.0 ng/ml (2–6 months post-transplant), 6.0–8.0 ng/ml (6–12 months), and 5.0–8.0 ng/ml (after 12 months). TTR categorization used a 60% cut-off.

### Data collection

Data were retrieved from the institutional CDW of the five participating medical centers (Supplementary Fig. S1, Supplemental Digital Content 1, http://links.lww.com/JS9/C773). To ensure relevance and consistency, investigators from all centers collaboratively defined the necessary variables and operational definitions. Custom extraction algorithms tailored to each CDW’s structure facilitated automated data collection, yielding a dataset encompassing recipient and donor demographics, transplant details, and follow-up information. A rigorous multi-step quality control process was applied, involving data cleansing, missing value imputation, inconsistency resolution, and duplicate removal, supplemented by manual verification and augmentation. Longitudinal individual patient data were collected from 1 year preoperatively to 1 or 6 years postoperatively, or until the last date of follow-up, according to the cohort definition. The list of variables is provided in Supplementary Methods, Supplemental Digital Content 1, http://links.lww.com/JS9/C773.

### Statistical analysis

Baseline characteristics were presented using descriptive statistics. The study population was grouped into seven tacrolimus trough level categories based on the tacrolimus trough level during the 2–4 months post-transplant period, and the between-group balance of baseline characteristics was checked using standardized mean differences. The Sankey diagram was used to visualize the changing patterns of the tacrolimus trough level over time.

Unadjusted survival analysis for the relationship between tacrolimus trough level and clinical outcomes was conducted using the standardized Cox proportional hazard model, with the periodic mean of tacrolimus trough level as the time-dependent covariate for all outcomes.

To control for confounding variables and obtain more accurate estimates of the effect of time-varying exposure (tacrolimus trough level) on the composite allograft outcome, we conducted an adjusted analysis using Cox MSM with stabilized weights calculated using the IPTW^28,29^. Tacrolimus levels at each time point are influenced not only by baseline patient characteristics but also by previous tacrolimus exposures and past clinical outcomes. The MSM approach allows for appropriate adjustment of these time-varying factors, providing more accurate causal estimates. This method adjusts for the confounding effect of imbalanced variables on both the probability that an individual will be allocated to the seven trough-level categories and the probability of the occurrence of the outcome. The stabilized weight at each time point consists of the product of the treatment weight and censoring weight. Treatment weights were calculated based on the inverse probability of each individual belonging to one of seven tacrolimus concentration categories at each observation time point, considering both time-dependent and time-independent covariates. Censoring weights were similarly calculated based on the probability of being censored at each time point. The covariates included for calculating stabilized weights were age, sex, previous dialysis months, immunosuppressant use other than tacrolimus, induction agent, desensitization, donor age, donor sex, and outcomes (rejection, renal dysfunction, and dnDSA) prior to the start of the cohort time and serum creatinine (time-dependent covariate). A detailed description of the method is provided in Supplementary Methods, Supplemental Digital Content 1, http://links.lww.com/JS9/C773.

We performed a standard Cox proportional hazards analysis for the association between the CV or TTR of tacrolimus trough level and clinical outcomes. In the unadjusted Cox analysis for the association of periodic mean tacrolimus trough levels and outcomes, as well as the Cox analyses for CV or TTR and outcomes, trough levels beyond the occurrence of the outcome of interest were excluded when calculating tacrolimus periodic mean, CV, or TTR.

Subgroup analyses were performed to explore potential variations in the association between tacrolimus exposure and primary endpoint within specific patient groups. These groups were defined by age (<18, 19–64, ≥65 years), diabetes, hypertension, donor type (living or deceased), desensitization status, and prior rejection history. Furthermore, for sensitivity analysis, we repeated the assessment of all outcomes using only tacrolimus levels below 25 ng/ml.

For all analyses, significance tests were two-sided, and a *P*-value of <0.05 was considered significant. Analyses were performed using SAS version 9.4 for Windows (SAS Institute, Cary, NC) and R (version 4.3.1). The current study has been reported in line with the STROCSS criteria^32^, Supplemental Digital Content 2, http://links.lww.com/JS9/C774.

## Results

**Figure 1:**
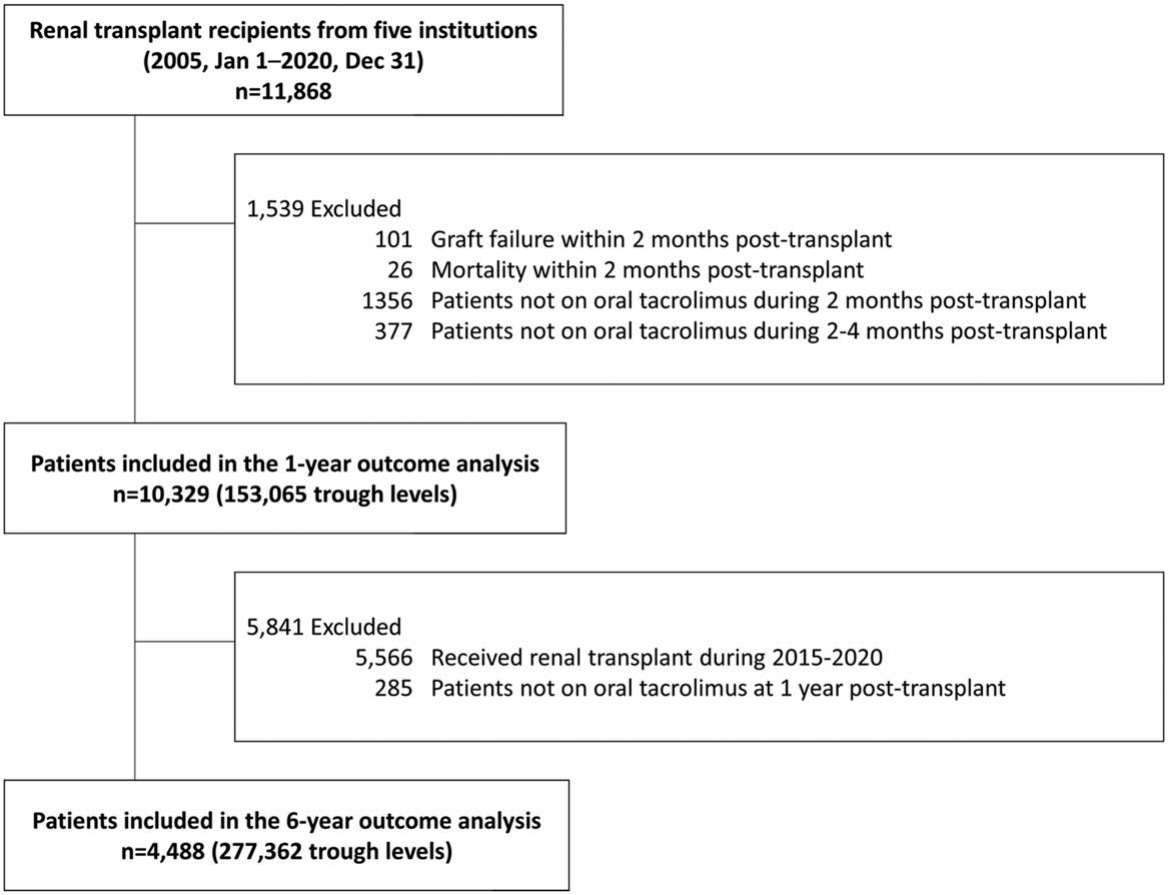
Flowchart of study patient selection.

A total of 11 868 patients underwent kidney transplants across five medical centers between 2005 and 2020 (Fig. 1). Of these, 10 329 patients, who contributed a total of serial 153 065 tacrolimus trough levels measurements, met the inclusion criteria for the primary 1-year outcome analysis. For the analysis of 6-year outcomes, a subset of 4488 patients who received transplants between 2005 and 2014 were included, contributing a total of 277 362 tacrolimus trough level measurements during the 2–6-year post-transplant period. Baseline characteristics are detailed in Table 1 (1-year cohort) and Supplementary Table S1, Supplemental Digital Content 1, http://links.lww.com/JS9/C773 (6-year cohort).

**Table 1:** Demographic and clinical characteristics of the 1-year analysis cohort (*n*=10 329).

		Tacrolimus trough level (ng/ml)								
	Total (*n*=10 329)	<3.0 (*n*=154)	3.0–3.9 (*n*=298)	4.0–4.9 (*n*=707)	5.0–5.9 (*n*=1222)	6.0–6.9 (*n*=1713)	7.0–7.9 (*n*=2106)	≥8.0 (*n*=4129)	*P* ^a^	SMD
Recipient characteristics										
Age, years, mean±SD	46.0±13.6	48.5±12.3	46.3±12.6	46.1±13.2	46.5±13.5	46.1±13.7	45.4±13.9	45.9±13.5	0.0658	2.10
Male sex, *n* (%)	5993 (58.0)	95 (61.7)	155 (52)	356 (50.4)	620 (50.7)	935 (54.6)	1189 (56.5)	2643 (64.0)	0.0000	8.87
BMI, kg/m^2^, mean±SD	23.0±25.5	24.5±13.7	22.8±4.6	22.9±5.9	22.6±4.7	22.7±5.2	23.8±55.3	22.9±5.8	0.7742	0.09
Hypertension, *n* (%)	6951 (67.3)	63 (40.9)	137 (46.0)	388 (54.9)	774 (63.3)	1174 (68.5)	1488 (70.7)	2927 (71.0)	0.0000	12.85
Diabetes mellitus, *n* (%)	2254 (21.8)	20 (13.0)	34 (11.4)	111 (15.7)	239 (19.6)	352 (20.6)	461 (21.9)	1037 (25.1)	0.0000	8.22
Primary etiology of ESRD, *n* (%)									0.0000	5.60
Diabetes	2045 (19.9)	35 (22.9)	45 (15.2)	123 (17.6)	219 (18.0)	309 (18.2)	407 (19.4)	907 (22.1)		
Hypertension	1001 (9.8)	11 (7.2)	23 (7.8)	51 (7.3)	118 (9.7)	181 (10.6)	201 (9.6)	416 (10.1)		
GN	1738 (16.9)	17 (11.1)	41 (13.9)	105 (15.0)	230 (18.9)	291 (17.1)	386 (18.4)	668 (16.3)		
PKD	561 (5.5)	4 (2.6)	19 (6.4)	49 (7.0)	67 (5.5)	98 (5.8)	112 (5.3)	212 (5.2)		
IgA nephropathy	1229 (12.0)	26 (17.0)	31 (10.5)	87 (12.4)	121 (10.0)	200 (11.8)	265 (12.6)	499 (12.2)		
Others	1361 (13.3)	8 (5.2)	32 (10.8)	107 (15.3)	199 (16.4)	256 (15)	300 (14.3)	459 (11.2)		
Unknown	2326 (22.7)	52 (34.0)	105 (35.5)	178 (25.4)	262 (21.5)	367 (21.6)	424 (20.2)	938 (22.9)		
Repeat transplant, *n* (%)	818 (7.9)	12 (7.8)	17 (5.7)	53 (7.5)	79 (6.5)	143 (8.4)	161 (7.6)	353 (8.6)	0.1951	2.13
Pre-transplant dialysis, *n* (%)	8468 (82.0)	106 (68.8)	225 (75.5)	532 (75.2)	993 (81.3)	1419 (82.8)	1752 (83.3)	3441 (83.5)	0.0000	6.41
Transplant characteristics										
PRA, %, mean±SD										
Class I	13.8±27.1	13.6±25.7	12.2±26.9	14.5±28.1	15.2±28.9	15.7±29.2	14.6±27.7	11.9±24.8	0.0003	3.58
Class II	13.4±27.2	16.2±30	14.4±29.1	14.4±28.6	14.9±29	14.5±28.3	14.1±27.8	11.7±25.3	0.0042	4.21
HLA-A/B/DR antigen mismatches, mean±SD	3.3±1.5	3.4±1.8	3.4±1.6	3.5±1.5	3.3±1.6	3.3±1.6	3.4±1.5	3.3±1.5	0.2513	1.94
Desensitization, *n* (%)	2457 (23.8)	53 (34.4)	70 (23.5)	212 (30.0)	327 (26.8)	453 (26.4)	495 (23.5)	847 (20.5)	0.0000	6.90
Pre-DSA positivity, *n* (%)	1177 (16.3)	27 (19.4)	25 (10.6)	76 (14.0)	145 (16.8)	222 (18.5)	270 (18.3)	412 (14.8)	0.0013	0.19
ABO incompatible, *n* (%)	1282 (14.8)	28 (21.4)	33 (14.5)	72 (15.3)	129 (14.7)	224 (16.7)	273 (15.0)	523 (13.8)	0.0777	1.22
Crossmatch positivity, *n* (%)	730 (7.1)	12 (7.8)	23 (7.7)	89 (12.6)	147 (12.0)	158 (9.2)	135 (6.4)	166 (4.0)	<0.0001	10.25
Induction therapy, *n* (%)									0.0000	0.64
None	268 (2.6)	7 (4.5)	5 (1.7)	8 (1.1)	17 (1.4)	39 (2.3)	35 (1.7)	157 (3.8)		
Basiliximab	7919 (76.7)	114 (74.0)	237 (79.5)	561 (79.3)	949 (77.7)	1318 (76.9)	1629 (77.4)	3111 (75.4)		
ATG	2105 (20.4)	33 (21.4)	56 (18.8)	138 (19.5)	254 (20.8)	351 (20.5)	434 (20.6)	839 (20.3)		
Others	35 (0.3)				2 (0.2)	5 (0.3)	8 (0.4)	20 (0.5)		
IS at 2 months post-transplant, *n* (%)										
Tacrolimus	7664 (74.2)	122 (79.2)	263 (88.3)	620 (87.7)	960 (78.6)	1245 (72.7)	1439 (68.3)	3015 (73.0)	0.0000	9.01
Once daily	245 (2.4)	17 (11.0)	33 (11.1)	53 (7.5)	61 (5.0)	32 (1.9)	28 (1.3)	21 (0.5)	0.0000	17.15
Twice daily	7426 (71.9)	106 (68.8)	230 (77.2)	568 (80.3)	900 (73.6)	1214 (70.9)	1412 (67)	2996 (72.6)	0.0000	3.05
Cyclosporine	29 (0.3)	7 (4.5)		3 (0.4)	3 (0.2)	4 (0.2)	6 (0.3)	6 (0.1)	0.0000	4.37
MMF or EC-MPA	6141 (59.5)	76 (49.4)	184 (61.7)	468 (66.2)	770 (63.0)	1086 (63.4)	1264 (60.0)	2293 (55.5)	0.0000	5.28
Steroid	4260 (41.2)	87 (56.5)	167 (56.0)	380 (53.7)	519 (42.5)	700 (40.9)	793 (37.7)	1614 (39.1)	0.0000	8.65
Others	363 (3.5)	23 (14.9)	17 (5.7)	32 (4.5)	40 (3.3)	60 (3.5)	58 (2.8)	133 (3.2)	0.0000	4.87
Donor information										
Age, years, mean±SD	44.7±13.3	45.9±13.7	45.1±13.7	45.3±13.2	45±13.1	44.8±13.3	45.1±13.3	44.1±13.3	0.0264	2.97
Male sex, *n* (%)	5349 (51.8)	65 (42.2)	141 (47.3)	362 (51.2)	616 (50.4)	909 (53.1)	1080 (51.3)	2176 (52.7)	0.0669	2.40
BMI, kg/m^2^, mean±SD	24.6±43.7	23.5±3.4	23.5±3.3	23.6±3.3	23.7±3.4	26.0±83.0	25.1±59.5	24.2±13.7	0.7945	0.42
Hypertension, *n* (%)	808 (11.5)	16 (11.1)	18 (6.7)	59 (9.4)	100 (10.4)	150 (12.4)	173 (12.6)	292 (12.1)	0.0363	3.34
Donor type, *n* (%)									0.0000	1.10
Living related	5145 (49.9)	88 (57.1)	131 (44.0)	363 (51.4)	611 (50.0)	831 (48.5)	1072 (51.0)	2049 (49.7)		
Living non-related	2056 (19.9)	38 (24.7)	87 (29.2)	173 (24.5)	270 (22.1)	353 (20.6)	360 (17.1)	775 (18.8)		
Deceased	3118 (30.3)	28 (18.1)	80 (26.9)	170 (24.1)	340 (27.8)	529 (30.9)	672 (31.9)	1299 (31.5)		

ATG, anti-thymocyte globulin; BMI, body mass index; DSA, donor-specific antibody; EC-MPA, enteric-coated mycophenolic acid; ESRD, end-stage renal disease; GN, glomerulonephritis; IgA, immunoglobulin A; IS, immunosuppression; MMF, mycophenolate mofetil; PKD, polycystic kidney disease; PRA, panel reactive antibodies; SD, standard deviation; SMD, standardized mean difference.

### Tacrolimus trough level variations over time

**Figure 2:**
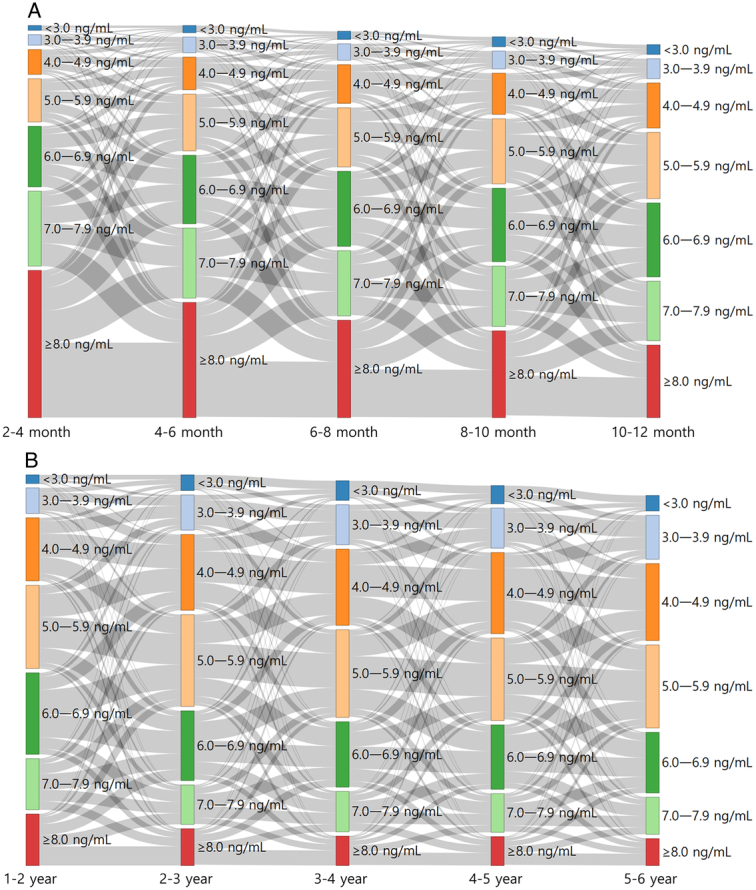
Sankey diagram of tacrolimus trough level changes. (A) Within 1 year post-transplant. (B) During 1–6 years post-transplant.

The most common tacrolimus trough level interval at 2 months post-transplant was ≥8.0 ng/ml (40.0%), followed by 7.0–7.9 ng/ml (20.4%) and 6.0–6.9 ng/ml (16.6%) (Supplementary Table S2, Supplemental Digital Content 1, http://links.lww.com/JS9/C773). The percentage of patients with a periodic mean tacrolimus trough level ≥6.0 ng/ml was over 60% until 10 months post-transplant, but decreased to about 50% thereafter and to less than 40% after 3 years post-transplant. The Sankey diagram (Fig. 2A, B) showed that periodic mean trough levels continued to oscillate across different tacrolimus concentration categories within 1-year and during 1–6 years post-transplant, manifesting the dynamic nature of tacrolimus concentration within this patient population.

### Tacrolimus trough levels and 1-year composite allograft outcomes

The primary endpoint, composite 1-year allograft outcome, was observed in 11.2% (1161/10 329) of the study population, with lower risks associated with tacrolimus trough levels of 5.0–5.9 ng/ml, 6.0–6.9 ng/ml, and 7.0–7.9 ng/ml. This composite included: BPR with an incidence of 8.8%; kidney dysfunction (eGFR <30 ml/min/1.73 m^2^) at 4.6%; dnDSA at 1.2%; and death-censored graft failure at 1.1% (Supplementary Table S3, Supplemental Digital Content 1, http://links.lww.com/JS9/C773).

**Figure 3:**
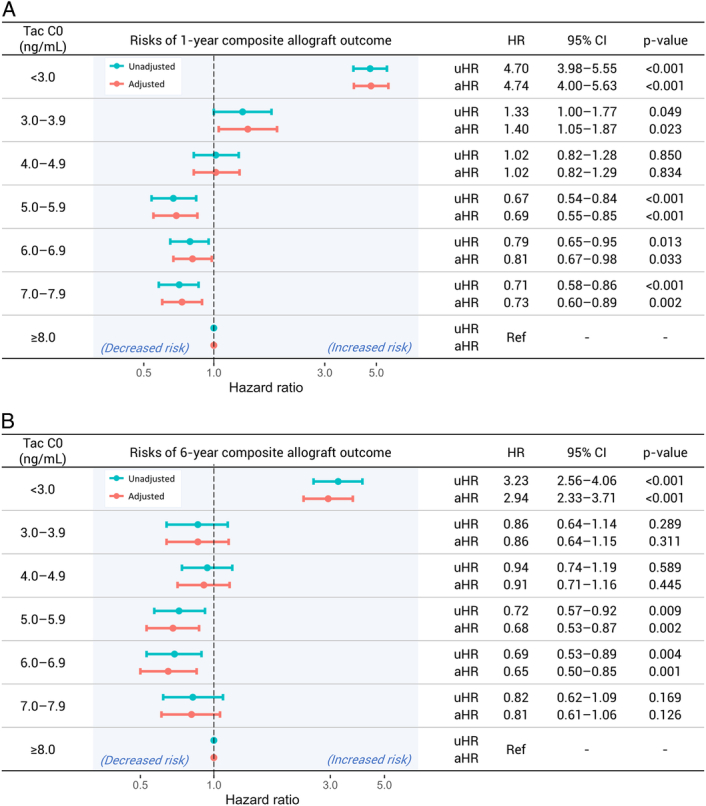
Risks of composite allograft outcome of biopsy-proven acute rejection, renal dysfunction, de novo donor-specific antibody development, and death-censored graft failure by periodic mean tacrolimus trough level. (A) Relative hazards of 1-year composite allograft outcome. (B) Relative hazards of 6-year composite allograft outcome. aHR, adjusted hazard ratio; C0, trough concentration; CI, confidence interval; HR, hazard ratio; uHR, unadjusted hazard ratio. For the unadjusted analysis, the *P*-value was calculated using the time-varying Cox proportional hazard model with periodic mean tacrolimus as a time-varying variable. The adjusted analysis incorporated the inverse probability of treatment weighting (IPTW) method with stabilized weight, which included sex; age; previous dialysis months; use of immunosuppressants other than tacrolimus; use of induction agents; desensitization; donor age; donor sex; donor-specific antibody at baseline; rejection/renal dysfunction status at baseline; and serum creatinine (time-dependent covariate) for the calculation of stabilized weights.

To evaluate the association between periodic mean tacrolimus trough levels and the primary outcomes, we employed two distinct analytical approaches: an unadjusted time-varying Cox proportional hazards model and the Cox MSM with IPTW for adjustment of confounding variables. The results for 1-year composite allograft outcomes were consistent across both methods (Fig. 3A). For the tacrolimus trough level categories 5.0–5.9 ng/ml, 6.0–6.9 ng/ml, and 7.0–7.9 ng/ml, adjusted hazard ratios (aHR) for experiencing the composite allograft outcome were significantly lower when compared to the ≥8.0 ng/ml group (aHR 0.69, 95% CI [confidence interval] 0.55–0.85, *P*<0.001; aHR 0.81, 95% CI 0.67–0.98, *P*=0.033; aHR 0.73, 95% CI 0.60–0.89, *P*=0.002, respectively). On the other hand, tacrolimus trough level categories <3.0 ng/ml and 3.0–3.9 ng/ml were associated with a higher risk of composite allograft outcomes (aHR 4.74, 95% CI 4.0–5.63, *P*<0.001; aHR 1.40, 95% CI 1.05–1.87, *P*=0.023, respectively).

Regarding the individual components of the composite allograft outcome, periodic mean tacrolimus trough levels of 5.0–5.9 ng/ml were associated with a reduced risk of developing BPR, and levels of 7.0–7.9 ng/ml were associated with a lower risk of kidney dysfunction (Supplementary Table S4, Supplemental Digital Content 1, http://links.lww.com/JS9/C773). Conversely, levels of 3.0–3.9 ng/ml and 4.0–4.9 ng/ml were associated with a higher risk of dnDSA development and death-censored graft failure. Notably, tacrolimus trough levels <3.0 ng/ml were linked to the elevated risk of all individual components.

The detrimental effects of periodic mean tacrolimus trough levels <3.0 ng/ml and the beneficial impacts of levels of 5.0–5.9 ng/ml, 6.0–6.9 ng/ml, and 7.0–7.9 ng/ml were consistent across most major subgroups (Supplementary Table S5, Supplemental Digital Content 1, http://links.lww.com/JS9/C773). However, certain subgroups (those aged 65 years and older, diabetic patients, living donor kidney recipients, and patients who experienced BPR before 2 months post-transplant) did not show statistically significant benefits from tacrolimus trough levels of 5.0–5.9 ng/ml, 6.0–6.9 ng/ml, and 7.0–7.9 ng/ml.

### Tacrolimus trough levels and 6-year composite allograft outcomes

The crude incidence of the composite allograft outcome during the 12–72 month period post-transplant was 23.1% (1037/4488; Supplementary Table S3, Supplemental Digital Content 1, http://links.lww.com/JS9/C773), and tacrolimus levels of 5.0–5.9 ng/ml and 6.0–6.9 ng/ml were associated with lower risks of 6-year allograft outcome.

Patients with periodic mean tacrolimus trough levels <3.0 ng/ml had an increased risk (aHR 2.94, 95% CI 2.33–3.71, *P*<0.001) of experiencing the composite outcome. Contrastingly, levels of 5.0–5.9 ng/ml and 6.0–6.9 ng/ml were associated with a significantly reduced risk of the composite outcome (aHR 0.68, 95% CI 0.53–0.84, *P*=0.002; aHR 0.65, 95% CI 0.50–0.85, *P*=0.001, respectively; Fig. 3B).

Further examination revealed that periodic mean tacrolimus tough levels ranging from 4.0 to 7.9 ng/ml were associated with a lower risk of kidney dysfunction (Supplementary Table S6, Supplemental Digital Content 1, http://links.lww.com/JS9/C773). Additionally, levels between 5.0 and 6.9 ng/ml showed reduced hazards for death-censored graft failure. Levels below 3.0 ng/ml correlated with elevated risks across all individual outcomes, including BPR, kidney dysfunction, dnDSA development, and death-censored graft failure.

### Tacrolimus trough levels and safety outcomes of infection, cardiovascular events, malignancy, and mortality

**Figure 4:**
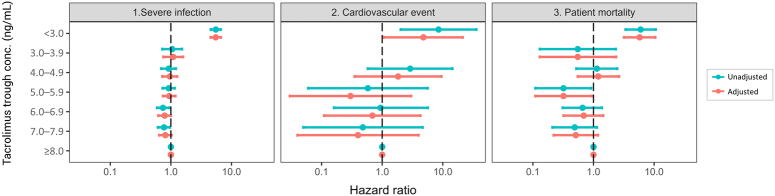
Association of tacrolimus trough levels and the risk of safety outcomes (severe infection, cardiovascular events, and mortality) 2–12 months post-transplant. All hazard ratios used ≥8 ng/ml as the reference. The adjusted analysis incorporated the inverse probability of treatment weighting (IPTW) method with stabilized weight and included the following covariates: sex; age; previous dialysis months; use of immunosuppressants other than tacrolimus; use of induction agents; desensitization; donor age; donor sex; and serum creatinine (time-dependent covariate) for the calculation of stabilized weights. Hazard ratios for cardiovascular events for tacrolimus trough concentrations 3.0–3.9 ng/ml were not estimable due to the small number of events.

The overall rates of severe infection, cardiovascular events, and mortality during the initial 2–12 months post-transplant were 8.2%, 0.1%, and 0.8%, respectively (Supplementary Table S3, Supplemental Digital Content 1, http://links.lww.com/JS9/C773). Patients with the lowest periodic mean tacrolimus trough levels (<3.0 ng/ml) faced significantly higher risks of severe infection (aHR 5.49, 95% CI 4.52–6.68, *P*<0.001), cardiovascular events (aHR 4.78, 95% CI 1.07–21.29, *P*=0.040), and mortality (aHR 5.78, 95% CI 3.19–10.48, *P*<0.001) when compared to those with levels ≥8.0 ng/ml (Fig. 4, Supplementary Table S7, Supplemental Digital Content 1, http://links.lww.com/JS9/C773). Conversely, the 5.0–5.9 ng/ml group had a reduced mortality risk (aHR 0.32, 95% CI 0.11–0.94, *P*=0.038).

**Figure 5:**
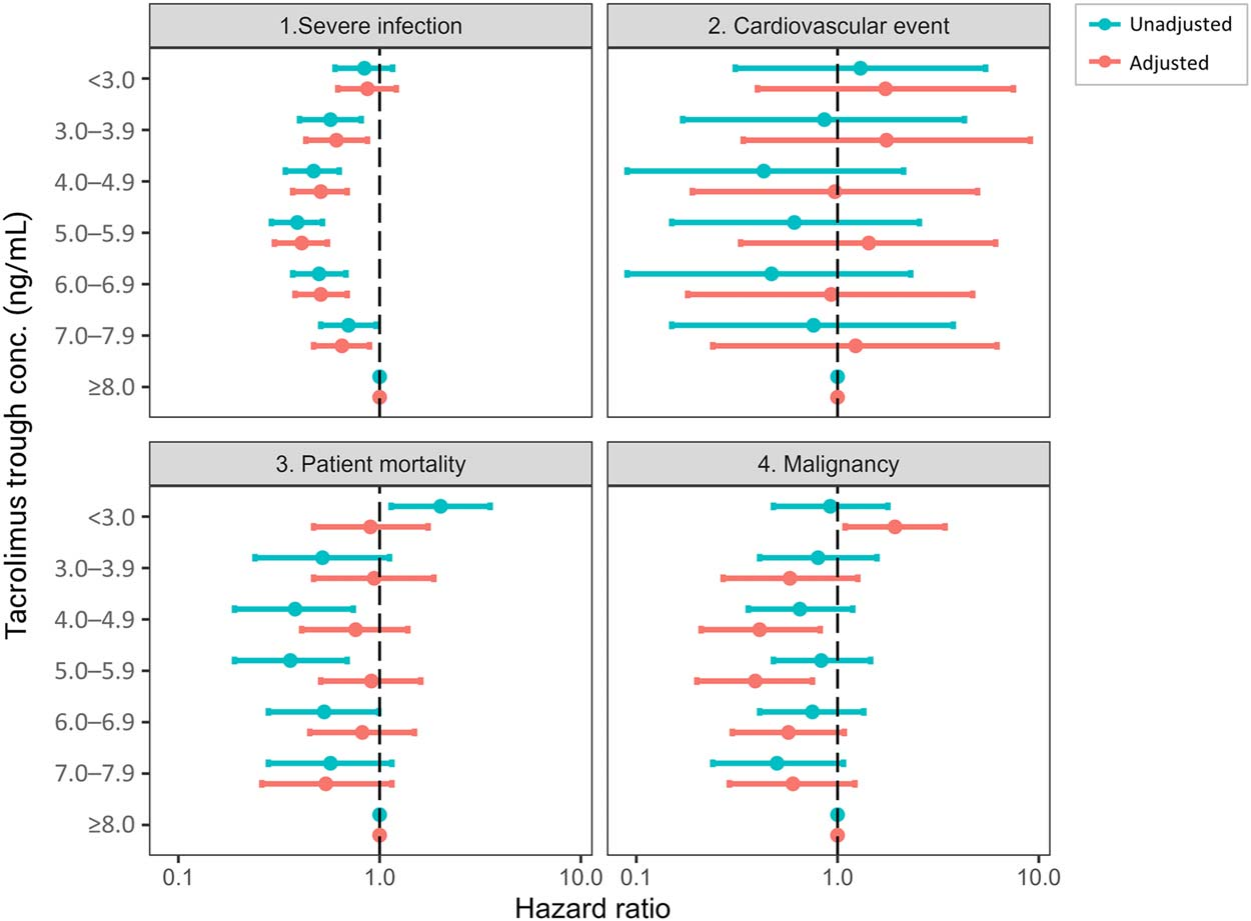
Association of tacrolimus trough levels and risk of safety outcomes (infection, cardiovascular events, malignancy, and mortality 12–72 months post-transplant. All hazard ratios used ≥8.0 ng/ml as the reference. The adjusted analysis incorporated the inverse probability of treatment weighting (IPTW) method with stabilized weight and included the following covariates: sex; age; previous dialysis months; use of immunosuppressants other than tacrolimus; use of induction agents; desensitization; donor age; donor sex; donor-specific antibody at baseline; rejection/kidney dysfunction status at baseline; and serum creatinine (time-dependent covariate) for the calculation of stabilized weights.

During the extended follow-up from 12–72 months post-transplant, the overall incidences of severe infection, cardiovascular events, malignancy, and mortality were 11.7%, 0.6%, 3.5%, and 2.9%, respectively (Supplementary Table S3, Supplemental Digital Content 1, http://links.lww.com/JS9/C773). Tacrolimus trough levels ranging from 3.0 to 7.9 ng/ml were correlated with a lower risk of severe infection compared to ≥8.0 ng/ml (Fig. 5, Supplementary Table S8, Supplemental Digital Content 1, http://links.lww.com/JS9/C773). For malignancy, levels below 3.0 ng/ml were associated with an elevated risk, while levels of 4.0–4.9 ng/ml and 5.0–5.9 ng/ml conferred reduced risk. No statistically significant associations were found for cardiovascular events or mortality.

### Tacrolimus coefficient of variability, and composite allograft and patient safety outcomes

Additional analysis of the tacrolimus CV and outcome variables showed that the quartile groups with a higher CV had a higher incidence of both 1-year and 6-year composite allograft outcomes, severe infection, mortality, and 6-year malignancy (Supplementary Table S9, Supplemental Digital Content 1, http://links.lww.com/JS9/C773). A multivariate Cox analysis revealed that the groups with lower CV quartiles were associated with a lower risk of composite allograft outcome, severe infection, and patient mortality at 1 and 6 years (Supplementary Table S10, Supplemental Digital Content 1, http://links.lww.com/JS9/C773). The lowest versus highest CV quartile group also showed a significantly lower risk of malignancy 2–6 years post-transplant. No associations were found between tacrolimus CV and cardiovascular events.

### Tacrolimus time in therapeutic range, and composite allograft and patient safety outcomes

Those who spent more time in predefined therapeutic range (high TTR; TTR ≥60%) showed a significantly lower incidence rate of composite allograft outcome at 1 and 6 years (Supplementary Table S11, Supplemental Digital Content 1, http://links.lww.com/JS9/C773), and were associated with a lower risk of developing composite allograft outcomes at 6 years (Supplementary Table S12, Supplemental Digital Content 1, http://links.lww.com/JS9/C773). Regarding patient safety outcome variables, the low TTR group was associated with a higher risk of severe infection at both 1 and 6 years and also higher mortality at 6 years (Supplementary Table S11, Supplemental Digital Content 1, http://links.lww.com/JS9/C773 and Supplementary S12, Supplemental Digital Content 1, http://links.lww.com/JS9/C773).

## Discussion

Our study utilized a comprehensive CDW dataset and advanced methods, including Cox MSM with IPTW, to define optimal post-transplant tacrolimus trough levels. Findings (Table 2) demonstrate that levels of 5.0–7.9 ng/ml (2–12 months post-transplant) and 5.0–6.9 ng/ml (2–6 years post-transplant are associated with reduced allograft risks). The suggested 2–6 year concentration ranges were also associated with lower incidences of severe infection and malignancy.

**Table 2:** Summary of the study findings: association between tacrolimus trough levels and 1-year and 6-year outcomes showing a significant increase (↑) or decrease (↓) of risk based on hazard estimates.

	Tacrolimus trough level (ng/ml)						
	<3.0	3.0–3.9	4.0–4.9	5.0–5.9	6.0–6.9	7.0–7.9	≥8.0
1-year outcome							
Allograft composite outcome	↑ (4.74)	↑ (1.40)	–	↓ (0.69)	↓ (0.81)	↓ (0.73)	Ref
BPR	↑ (2.97)	–	–	↓ (0.71)	–	–	Ref
Kidney dysfunction	↑ (6.85)	↑ (1.55)	–	↓ (0.68)	–	↓ (0.58)	Ref
dnDSA	↑ (14.32)	↑ (5.47)	↑ (4.31)	–	–	–	Ref
DCGF	↑ (12.42)	↑ (4.80)	↑ (2.50)	–	–	–	Ref
Severe infection	↑ (5.49)	–	–	–	–	–	Ref
MACE	↑ (4.78)	–	–	–	–	–	Ref
Mortality	↑ (5.78)	–	–	↓ (0.32)	–	–	Ref
2–6-year outcome							
Allograft composite outcome	↑ (2.94)	–	–	↓ (0.68)	↓ (0.65)	–	Ref
BPR	↑ (2.66)	–	–	–	–	–	Ref
Kidney dysfunction	↑ (2.46)	↓ (0.65)	↓ (0.56)	↓ (0.42)	↓ (0.46)	↓ (0.52)	Ref
dnDSA	↑ (2.67)	–	–	–	–	–	Ref
DCGF	↑ (3.27)	–	–	↓ (0.58)	↓ (0.53)	–	Ref
Severe infection	–	↓ (0.61)	↓ (0.51)	↓ (0.41)	↓ (0.51)	↓ (0.65)	Ref
MACE	–	–	–	–	–	–	Ref
Malignancy	↑ (1.93)	–	–	↓ (0.41)	↓ (0.39)	–	Ref
Mortality	–	–	–	–	–	–	Ref

Hazard ratios (values in brackets) are derived from adjusted analysis using the inverse probability of treatment weighting (IPTW) method with stabilized weights.

Dashes (–) indicate hazard ratios that were not statistically significant

BPR, biopsy-proven rejection; DCGF, death-censored graft failure; dnDSA, de novo donor-specific antibody; MACE, major adverse cardiovascular events; Ref, references.

While tacrolimus has become the cornerstone of immunosuppressive therapy, its ideal target trough level for balancing efficacy and toxicity remains controversial^4,5,10^. In the ELITE-Symphony trial, patients maintained tacrolimus trough concentrations of 5.0–10.0 ng/ml (mean 6.0–8.0 ng/ml) throughout the first year, resulting in improved outcomes when compared to sirolimus or cyclosporine-based regimens^3^. However, a pooled analysis of three randomized trials, including the Symphony trial, failed to establish an optimal trough level, potentially due to limited sampling at only five discrete time points early post-transplant^33^. Our study adds to this dialogue by suggesting a lower limit of 5.0 ng/ml tacrolimus during the 2–12 months post-transplant period for both composite and individual graft outcomes. Our limit is consistent with those suggested by Wiebe *et al*.^34^, who showed that a greater proportion of patients developing HLA DR/DQ dnDSA had tacrolimus levels below 5.0 ng/ml. In contrast, Davis *et al*.^35^ recommended a higher limit, suggesting that a tacrolimus trough level of less than 8.0 ng/ml within the first 6 or 12 months post-transplant led to higher risks of dnDSA development by 6 and 12 months. The differences may be attributed to variations in study populations and analysis methodologies. Instead of dichotomizing or quartilizing the cohort based on mean tacrolimus level, as in Davis *et al*., our approach offered a more granular analysis by comparing outcomes across multiple tacrolimus concentration groups based on a 2-month periodic mean. This was made possible by the sample size, which exceeded 10 000 patients and provided 430 427 serial tacrolimus concentrations.

Regarding the long-term impact of tacrolimus, there has been a paucity of studies examining optimal concentrations beyond the first year post-transplant. Our study fills this gap by showing that tacrolimus trough levels 5.0–5.9 ng/ml and 6.0–6.9 ng/ml were significantly beneficial compared to levels above 8.0 ng/ml. This contrasts with Unagami *et al*.^36^, who did not find significant differences in dnDSA or kidney function among patients with different tacrolimus trough levels up to 7 years post-transplant. Their study, however, was limited by its small sample size in the low (≤4.0 ng/ml) and high (>6.0 ng/ml) trough-level groups and its inability to capture the dynamic changes in tacrolimus concentrations. In support of our findings, another study using CTS registry data highlighted the potential adverse effects of maintaining low tacrolimus levels in the long term^23^. This study, which evaluated 6638 patients, showed that a tacrolimus trough level below 4.0 ng/ml by the third year was associated with significantly lower graft survival 4–6 years post-transplant compared to patients with higher trough levels.

Our analysis revealed a non-linear relationship between tacrolimus trough levels and the risk of individual components of the composite allograft outcomes. The main drivers for decreased risk within the 5.0–7.9 ng/ml concentration range for 1-year outcomes were rejection and kidney dysfunction. For 2–6 years graft outcome, the 5.0–6.9 ng/ml range proved beneficial, primarily due to reduced kidney dysfunction and graft failure. Notably, lower rates of kidney dysfunction in these concentration ranges, especially significant during the 2–6-year period, likely underscore the benefits of lower tacrolimus concentrations in mitigating drug-induced nephrotoxicity. This observation aligns with histological evidence suggesting that chronic changes are induced by long-term exposure to calcineurin inhibitors^37^.

Although there is significant concern about the long-term use of immunosuppressive medication for its systemic effects, it remains unclear whether certain tacrolimus levels are associated with adverse non-graft outcomes. Our results reveal a trend of lower risks of severe infection in the 6.0–7.9 ng/ml range in the first year (not significant) and significantly lower risk in the 3.0–7.9 ng/ml ranges during 2–6 years of transplant compared to those over 8.0 ng/ml. On the other hand, we observed an unexpectedly high risk of severe infection and mortality in the less than 3.0 ng/ml group. This may reflect the inclusion of patients who had been targeted for lower tacrolimus concentrations due to outpatient-managed infections before experiencing a severe infection requiring hospitalization.

Regarding malignancy risk, our adjusted analysis indicates that tacrolimus levels within the 4.0–4.9 ng/ml and 5.0–5.9 ng/ml ranges are associated with lower risks of malignancy compared to levels exceeding 8.0 ng/ml during 2–6 years post-transplant. This finding complements a nested case–control study that examined the association between early tacrolimus levels (at 6 and 12 months post-transplant) and the subsequent risk of malignancy after 3 years^38^, suggesting that higher tacrolimus levels may increase the risk of malignancy. Unlike the case–control study, our analysis specifically examines the relationship between tacrolimus levels during the 2–6 years post-transplant period and the incidence of malignancy within the same timeframe, further suggesting that long-term tacrolimus levels may also be important in determining individual malignancy risk.

A large patient population is indispensable for rigorous evaluation of the association between tacrolimus concentration and long-term outcomes, especially given the relatively lower frequency of events like dnDSA and rejection after the first year. While available real-world data (RWD) from registry studies serve this purpose, they are often limited by periodic tacrolimus concentration measurements at fixed time intervals^22,23^. Emerging evidence suggests that time spent at low tacrolimus levels and the variability of tacrolimus concentration are crucial determinants of outcomes^24,25^, so continuous data capture is paramount. Our study successfully amalgamated the benefits of both large sample size and continuous data by conducting a multicenter investigation leveraging institutional CDW platforms. This entailed intricate data mapping, meticulous curation of data extraction algorithms tailored to each participating institution, and rigorous quality assessments. Building on the existing RWD literature, our study offers a more detailed analysis with larger sample size and the use of consecutive tacrolimus concentration measurements.

Advancements in data integration and extraction technologies have made real-world big data, including electronic health record data, claims data, and registry data, more readily available. These data sources have the capability to produce valid and impartial real-world evidence, offering substantial reductions in cost and time compared to controlled trials^39,40^. Additionally, RWD can provide insights into treatment effects in scenarios where randomized controlled trials are not feasible due to technical, ethical, or economic reasons. However, despite these advantages of RWD, careful design and appropriate statistical methods are essential for drawing valid causal inferences^39^. As emphasized by Hernán and Robins’ target trial framework, rigorous approaches are necessary to define causal questions akin to those addressed by controlled trials, specifying a hypothetical protocol and planning meticulously on how to mimic such studies using RWD^41^. Our study demonstrates the potential of these approaches in answering complex causal questions, such as the optimal tacrolimus trough level range for graft outcomes and safety after renal transplantation from RWD. By utilizing a multicenter CDW-based database and employing the Cox MSM with IPTW, we aimed to emulate the essential features of controlled trials and estimate causal effects while addressing potential biases from time-varying confounders.

Our methodology of IPTW with stabilized weights in a time-varying analysis to mitigate confounding also deserves mention. The inherent challenge in studying the effects of tacrolimus levels on outcomes is their intentional modulation based on various covariates; for example, a history of acute rejection within the first 2 months post-transplant could influence both the risk of subsequent rejection (outcome) and the tacrolimus concentration a clinician target (exposure). By incorporating both baseline and time-dependent covariates into the calculation of stabilized weights, we aimed to control for the confounding effects of these variables on the relationship between tacrolimus trough levels and relevant outcomes. This methodological rigor facilitated balanced comparisons between different treatment groups and mitigated bias from potential confounders. Additionally, the Cox MSM with IPTW helped address attrition bias by accounting for differential loss to follow-up. Overall, the utilization of stabilized weights within the IPTW framework reinforced the methodological integrity of our study, minimizing biases and enhancing the reliability of our findings regarding the association between tacrolimus trough levels and post-transplant outcomes.

Our study has several limitations. Despite the substantial sample size, the study was limited by its retrospective design and the variability in data structures across participating institutions. The study also had to forgo the inclusion of viral infection status as a time-varying covariate due to significant data gaps caused by differences in surveillance protocols. While the large sample size enhanced the generalizability of our findings, it is essential to note that the results may not extend to settings with different medical resources and protocols, as the participating centers were all large tertiary care hospitals. In addition, the ethnic homogeneity of our study could affect the generalizability of our findings to non-Asian populations. Previous studies have suggested that pharmacogenetic differences may influence the metabolism and efficacy of tacrolimus across different ethnic groups^42^. Therefore, further studies are needed to extrapolate our results to populations with diverse ethnic backgrounds. Lastly, the extended study duration from 2005 to 2020 could introduce potential time effects related to changes in baseline patient characteristics and post-transplant management protocols. Although our analysis adjusted for these factors, there may still be residual time-related influences that were not fully accounted for.

## Conclusion

This multicenter study provides evidence for optimal tacrolimus trough levels during the 2–12 and 12–72 months post-transplantation. Our findings suggest that maintaining tacrolimus levels within 5.0–7.9 ng/ml for the first year and 5.0–6.9 ng/ml for years 2–6 correlates with high graft survival and optimal safety outcomes. Optimizing tacrolimus use may improve graft and patient outcomes, enhance overall renal transplantation success rates, and extend benefits to a greater number of patients. This is critical given the ongoing imbalance between organ demand and supply. Improving graft survival and reducing complications will directly support the goal of transplant surgeons to treat end-stage organ disease and maximize the therapeutic potential of transplantation.

## Ethical approval

The study protocol was approved by the institutional review boards of the five participating centers in Korea: Seoul National University Hospital (IRB No. 2107-196-1237), Asan Medical Center (IRB No. 2022-0139), Severance Hospital (IRB No. 4-2021-1377), Seoul St. Mary’s Hospital (IRB No. KC21WIDI0910), and Samsung Medical Center (IRB No. 2022-03-133-002).

## Consent

Informed consent was waived due to the retrospective noninterventional design of the study. This was approved by the institutional review boards of the five participating centers.

## Source of funding

This study was funded by Astellas Pharma, Inc. (Grant No. FY20-2963).

## Author contribution

A.H. and S.M.: conceptualization; A.H., H.K., Y.H.K., J.L., K.H.H., K.W.L., J.B.P., S.K.M., S.C.P., and S.M.: data curation; A.J.J. and J.L.: formal analysis; S.M., J.L., Y.K., and M.S.: funding acquisition; A.J.J., J.L., A.H., and S.M.: methodology; A.H.: writing – original draft; H.K., Y.H.K., J.L., K.H.H., K.W.L., J.B.P., S.K.M., S.C.P., A.J.J., J.L., and J.L., Y.K., M.S., and S.M.: writing – review and editing; A.J.J. and A.H.: visualization.

## Conflicts of interest disclosure

Jeongyun Lee and Younghye Kim are employees of Astellas Pharma, Korea; Mohamed Soliman is an employee of Astellas Pharma Singapore Pte Ltd, Singapore.

## Research registration unique identifying number (UIN)

*Clinical Trial Registration:* ClinicalTrials.gov; number NCT06348446.

## Guarantor

Sangil Min, Department of Surgery, Seoul National University College of Medicine, 101 Daehak-ro, Jongro-gu, Seoul 03080, Republic of Korea, Tel.: 82 2 2072 2330; e-mail: surgeonmsi@gmail.com.

## Data availability statement

The data that support the findings of this study are available from the corresponding author upon reasonable request. However, due to the nature of the data and the policies of the institutional review board (IRB), some restrictions may apply to the availability of these data.

## Provenance and peer review

Not commissioned, externally peer-reviewed.

## Presentation

None.

## Supplementary Material

SUPPLEMENTARY MATERIAL

## References

[1] Chaudhry D Chaudhry A Peracha J . Survival for waitlisted kidney failure patients receiving transplantation versus remaining on waiting list: systematic review and meta-analysis. BMJ 2022;376:e068769. 35232772 10.1136/bmj-2021-068769 PMC8886447

[2] Wang Y Hemmelder MH Bos WJW . Mapping health-related quality of life after kidney transplantation by group comparisons: a systematic review. Nephrol Dial Transplant 2021;36:2327–2339. 34338799 10.1093/ndt/gfab232 PMC8643597

[3] Lim MA Kohli J Bloom RD . Immunosuppression for kidney transplantation: Where are we now and where are we going? Transplant Rev (Orlando) 2017;31:10–17. 28340885 10.1016/j.trre.2016.10.006

[4] Lentine KL Smith JM Miller JM . OPTN/SRTR 2021 Annual Data Report: Kidney. Am J Transplant 2023;23:S21–S120. 37132350 10.1016/j.ajt.2023.02.004 PMC9970360

[5] Australia & New Zealand Dialysis & Transplant Registry (ANZDATA) . ANZDATA 45th Annual Report 2022 Accessed 12 December 2023. https://www.anzdata.org.au/report/anzdata-45th-annual-report-2022-data-to-2021/

[6] Szumilas K Wilk A Wiśniewski P . Current status regarding immunosuppressive treatment in patients after renal transplantation. Int J Mol Sci 2023;24:10301. 37373448 10.3390/ijms241210301 PMC10298917

[7] Nankivell BJ PʼNg CH OʼConnell PJ . Calcineurin inhibitor nephrotoxicity through the lens of longitudinal histology: comparison of cyclosporine and tacrolimus eras. Transplantation 2016;100:1723–1731. 27306529 10.1097/TP.0000000000001243

[8] Karolin A Genitsch V Sidler D . Calcineurin inhibitor toxicity in solid organ transplantation. Pharmacology 2021;106:347–355. 34130291 10.1159/000515933

[9] Farouk SS Rein JL . The many faces of calcineurin inhibitor toxicity–What the FK? Adv Chronic Kidney Dis 2020;27:56–66. 32147003 10.1053/j.ackd.2019.08.006 PMC7080294

[10] Andrews LM Li Y De Winter BCM . Pharmacokinetic considerations related to therapeutic drug monitoring of tacrolimus in kidney transplant patients. Expert Opin Drug Metab Toxicol 2017;13:1225–1236. 29084469 10.1080/17425255.2017.1395413

[11] Kershner RP Fitzsimmons WE . Relationship of FK506 whole blood concentrations and efficacy and toxicity after liver and kidney transplantation. Transplantation 1996;62:920–926. 8878385 10.1097/00007890-199610150-00009

[12] Laskow DA Vincenti F Neylan JF . An open-label, concentration-ranging trial of FK506 in primary kidney transplantation: a report of the United States Multicenter FK506 Kidney Transplant Group. Transplantation 1996;62:900–905. 8878381 10.1097/00007890-199610150-00005

[13] McMaster P Mirza DF Ismail T . Therapeutic drug monitoring of tacrolimus in clinical transplantation. Therapeutic Drug Monitoring 1995;17:602–605. 8588228 10.1097/00007691-199512000-00010

[14] Ekberg H Bernasconi C Tedesco-Silva H . Calcineurin inhibitor minimization in the Symphony study: observational results 3 years after transplantation. Am J Transplant 2009;9:1876–1885. 19563339 10.1111/j.1600-6143.2009.02726.x

[15] Aktürk S Erdoğmuş Ş Kumru G . Average tacrolimus trough level in the first month after transplantation may predict acute rejection. Transplant Proc 2017;49:430–435. 28340806 10.1016/j.transproceed.2017.02.011

[16] Gaynor JJ Ciancio G Guerra G . Lower tacrolimus trough levels are associated with subsequently higher acute rejection risk during the first 12 months after kidney transplantation. Transpl Int 2016;29:216–226. 26442829 10.1111/tri.12699

[17] Israni AK Riad SM Leduc R . Tacrolimus trough levels after month 3 as a predictor of acute rejection following kidney transplantation: a lesson learned from DeKAF Genomics. Transpl Int 2013;26:982–989. 23879408 10.1111/tri.12155 PMC3787982

[18] O’Seaghdha CM McQuillan R Moran AM . Higher tacrolimus trough levels on days 2-5 post-renal transplant are associated with reduced rates of acute rejection. Clin Transplant 2009;23:462–468. 19681975 10.1111/j.1399-0012.2009.01021.x

[19] Richards KR Hager D Muth B . Tacrolimus trough level at discharge predicts acute rejection in moderately sensitized renal transplant recipients. Transplantation 2014;97:986–991. 24784360 10.1097/TP.0000000000000149

[20] Yin S Song T Jiang Y . Tacrolimus trough level at the first month may predict renal transplantation outcomes among living Chinese kidney transplant patients: a propensity score-matched analysis. Ther Drug Monit 2019;41:308–316. 31083041 10.1097/FTD.0000000000000593 PMC6553958

[21] Borobia AM Romero I Jimenez C . Trough tacrolimus concentrations in the first week after kidney transplantation are related to acute rejection. Ther Drug Monit 2009;31:436–442. 19494792 10.1097/FTD.0b013e3181a8f02a

[22] Jung H-Y Seo MY Jeon Y . Tacrolimus trough levels higher than 6 ng/mL might not be required after a year in stable kidney transplant recipients. PLoS One 2020;15:e0235418. 32614859 10.1371/journal.pone.0235418 PMC7332007

[23] Süsal C Döhler B . Late intra-patient tacrolimus trough level variability as a major problem in kidney transplantation: a Collaborative Transplant Study Report. Am J Transplant 2019;19:2805–2813. 30859672 10.1111/ajt.15346

[24] Park Y Lee H Eum SH . Intrapatient variability in tacrolimus trough levels over 2 years affects long-term allograft outcomes of kidney transplantation. Front Immunol 2021;12:746013. 34659243 10.3389/fimmu.2021.746013 PMC8514869

[25] Davis S Gralla J Klem P . Tacrolimus intrapatient variability, time in therapeutic range, and risk of de novo donor-specific antibodies. Transplantation 2020;104:881–887. 32224815 10.1097/TP.0000000000002913

[26] Hamoud A Hashim AS Awadh WA . Clinical data warehouse: a review. Iraqi J Comput Inform 2018;44:16–26.

[27] Prather JC Lobach DF Goodwin LK . Medical data mining: knowledge discovery in a clinical data warehouse. Proc AMIA Annu Fall Symp 1997:101–105. 9357597 PMC2233405

[28] Hernán MA Brumback B Robins JM . Marginal structural models to estimate the causal effect of zidovudine on the survival of HIV-positive men. Epidemiology 2000;11:561–570. 10955409 10.1097/00001648-200009000-00012

[29] Xu Q Przepiorka D . Using marginal structural models to analyze the impact of subsequent therapy on the treatment effect in survival data: simulations and clinical trial examples. Pharm Stat 2021;20:1088–1101. 33908174 10.1002/pst.2127

[30] Schmitt L Speckman J Ansell J . Quality assessment of anticoagulation dose management: comparative evaluation of measures of time-in-therapeutic range. J Thromb Thrombolysis 2003;15:213–216. 14739631 10.1023/B:THRO.0000011377.78585.63

[31] Rosendaal FR Cannegieter SC van der Meer FJ . A method to determine the optimal intensity of oral anticoagulant therapy. Thromb Haemost 1993;69:236–239. 8470047

[32] Mathew G Agha R Albrecht J . STROCSS 2021: strengthening the reporting of cohort, cross-sectional and case–control studies in surgery. Int J Surg 2021;96:106165. 34774726 10.1016/j.ijsu.2021.106165

[33] Bouamar R Shuker N Hesselink DA . Tacrolimus predose concentrations do not predict the risk of acute rejection after renal transplantation: a pooled analysis from three randomized-controlled clinical trials(†). Am J Transplant 2013;13:1253–1261. 23480233 10.1111/ajt.12191

[34] Wiebe C Rush DN Nevins TE . Class II eplet mismatch modulates tacrolimus trough levels required to prevent donor-specific antibody development. J Am Soc Nephrol 2017;28:3353–3362. 28729289 10.1681/ASN.2017030287 PMC5661295

[35] Davis S Gralla J Klem P . Lower tacrolimus exposure and time in therapeutic range increase the risk of de novo donor-specific antibodies in the first year of kidney transplantation. Am J Transplant 2018;18:907–915. 28925597 10.1111/ajt.14504 PMC5858995

[36] Unagami K Ishida H Furusawa M . Influence of a low-dose tacrolimus protocol on the appearance of de novo donor-specific antibodies during 7 years of follow-up after renal transplantation. Nephrol Dial Transplant 2021;36:1120–1129. 33280052 10.1093/ndt/gfaa258 PMC8160958

[37] Nankivell BJ Borrows RJ Fung CLS . Calcineurin inhibitor nephrotoxicity: longitudinal assessment by protocol histology. Transplantation 2004;78:557–565. 15446315 10.1097/01.tp.0000128636.70499.6e

[38] Lichtenberg S Rahamimov R Green H . The incidence of post-transplant cancer among kidney transplant recipients is associated with the level of tacrolimus exposure during the first year after transplantation. Eur J Clin Pharmacol 2017;73:819–826. 28342067 10.1007/s00228-017-2234-2

[39] Liu F Panagiotakos D . Real-world data: a brief review of the methods, applications, challenges and opportunities. BMC Med Res Methodol 2022;22:287. 36335315 10.1186/s12874-022-01768-6 PMC9636688

[40] Qiao H Chen Y Qian C . Clinical data mining: challenges, opportunities, and recommendations for translational applications. J Transl Med 2024;22:185. 38378565 10.1186/s12967-024-05005-0 PMC10880222

[41] Hernán MA Robins JM . Using big data to emulate a target trial when a randomized trial is not available. Am J Epidemiol 2016;183:758–764. 26994063 10.1093/aje/kwv254 PMC4832051

[42] Tang JT Andrews LM van Gelder T . Pharmacogenetic aspects of the use of tacrolimus in renal transplantation: recent developments and ethnic considerations. Expert Opin Drug Metab Toxicol 2016;12:555–565. 27010623 10.1517/17425255.2016.1170808

