# Supplementary material for: Optimum tacrolimus trough levels for enhanced graft survival and safety in kidney transplantation: a retrospective multicenter real-world evidence study
Source: Int J Surg. 2024 Jun 17;110(10):6711–22. doi: 10.1097/JS9.0000000000001800 (PMC11486932; doi:10.1097/JS9.0000000000001800)

Supplemental Digital Content

**<Supplementary Methods>**

1. Definitions of major outcomes

2. Statistical methods for the analysis of the primary endpoint

3. Database variables

**<Supplementary Tables>**

**Table S1.** Demographic and clinical characteristics of the 6-year analysis cohort (n=4,488)

**Table S2.** Periodic mean tacrolimus trough levels 2–12 and 12–72 months post-transplant

**Table S3.** Crude incidence of primary and secondary transplant outcomes by baseline tacrolimus trough level

**Table S4.** Relative hazards of biopsy-proven rejection, renal dysfunction, de novo donor-specific antibodies, and death-censored graft failure 2–12 months post-transplant according to periodic mean tacrolimus trough range

**Table S5.** Relative hazards of the 1-year composite allograft outcome according to periodic mean tacrolimus trough range in pre-defined subgroups

**Table S6.** Relative hazards of biopsy-proven rejection, kidney dysfunction, de novo donor-specific antibodies, death-censored graft failure 12–72 months post-transplant according to periodic mean tacrolimus trough range

**Table S7.** Relative hazards of safety outcomes of severe infection, cardiovascular events, and patient mortality 2–12 months post-transplant according to periodic mean tacrolimus trough range

**Table S8.** Relative hazards of safety outcomes of severe infection, cardiovascular events, and patient mortality 12–72 months post-transplant according to yearly mean tacrolimus trough range

**Table S9.** Crude incidence of allograft and patient outcomes 2–12 and 12–72 months post-transplant categorized by quartiles of tacrolimus trough level coefficient of variability

**Table S10.** Multivariate Cox analysis of the risk of allograft and patient outcomes 2–12 and 12–72 months post-transplant across different tacrolimus trough level coefficient of variability groups

**Table S11.** Crude incidence of allograft and patient outcomes 2–12 and 12–72 months post-transplant categorized by tacrolimus time in therapeutic range groups

**Table S12.** Multivariate Cox analysis of the risk of allograft and patient outcomes 2–12 and 12–72 months post-transplant across high and low time in therapeutic range groups

**<Supplementary Figure>**

**Figure S1.** Data collection scheme illustrating the use of clinical data warehouses (CDW) from the five participating centers

**1. Definitions of major outcomes**

- **Biopsy-proven rejection (BPR)** - Pathologic diagnosis of rejection made by Banff criteria 2015. Rejection not based on Banff criteria 2015 was reassessed based on individual Banff lesion grading score. Episodes of rejection identified in protocol biopsies were also classified as biopsy-proven rejections.
- **Kidney dysfunction** - Defined as more than three consecutive estimated glomerular filtration rates (eGFR) less than 30 mL/min/1.73m². The eGFR is calculated using the MDRD equation for individuals aged ≥18 years or the Schwartz formula for those <18 years of age.
- **de novo donor specific antibody** - The novel presence of antibodies against donor HLA antigens at the HLA-A, -B, -DR, and -DQ loci with an absolute mean fluorescence intensity (MFI) of greater than 500.
- **Graft failure** - The necessity to return to dialysis dependence, defined as the need for dialysis for more than three months or until the last follow-up if less than three months have elapsed, re-transplantation, or nephrectomy.
- **Severe infection** - Any infection necessitating hospital admission. Identification was conducted through ICD codes for admission episodes derived from Koo et al.^1^, alongside a history of antibiotic usage, and was re-confirmed by manual review of electronic medical records.
- **Major cardiovascular event** - Hospitalization due to a primary diagnosis of myocardial infarction or ischemic stroke/transient ischemic attack, or the occurrence of a procedural charge for percutaneous coronary intervention or coronary artery bypass graft surgery. The screening for myocardial infarction or ischemic stroke/transient ischemic attack employed ICD-10 codes I219, I214, I210, I211, I212, I213, I220, I221, I228, I249, I200, I661-3, I660, I664, I668, I662, I669, I64X, I651-3, I658-9, I650, G458-9, I636, I632, I630, I634, I631, I633, I639, I635, I638, I693-4. Ambiguous cases were clarified through manual electronic medical chart review.
- **Malignancy -** Identified using ICD-10 codes C00-C96. Cases were individually reviewed to exclude any malignancies diagnosed prior to the transplant.

^1^ Koo BS, Lim YC, Lee MY, Jeon JY, Yoo HJ, Oh IS, Shin JY, Kim TH. The risk factors and incidence of major infectious diseases in patients with ankylosing spondylitis receiving tumor necrosis factor inhibitors. Mod Rheumatol. 2021 Nov;31(6):1192-1201.

**2. Statistical methods for the analysis of the primary endpoint**

The primary endpoint was the combined incidence of either biopsy proven acute rejection, renal dysfunction (eGFR<30), development of de novo DSA, or death censored graft failure that occurred within 1-year from the latest transplant. To assess the association between time varying periodic mean of tacrolimus trough level during 2-12 mo. post-transplant and survival time of 1-year composite allograft outcome, an analytical approach considering time varying confounding affected by past exposure was used.Specifically, the periodic mean of tacrolimus trough level was categorized into <3ng/ml, 3-3.9 ng/ml, 4-4.9ng/ml, 5-5.9ng/ml, 6-6.9ng/ml, 7-7.9ng/ml, and ≥8ng/ml (i.e., seven categories) and included as an exposure variable in the model because a unit increment in the tacrolimus trough concentration may not significantly affect the risk for the event. The following figure shows a possible example of the seven categories assigned to each time periods.

|  | **2-4 mo.** | **4-6 mo.** | **6-8 mo.** | **8-10 mo.** | **10-12 mo.** |
| --- | --- | --- | --- | --- | --- |
| **Patient 1** | ‘3-3.9 ng/ml’ | ‘7-7.9ng/ml’ | ‘6-6.9ng/ml’ | ‘4-4.9ng/ml’ | ‘<3ng/ml’ |
| **Patient 2** | ‘<3ng/ml’ | ‘6-6.9ng/ml’ | ‘4-4.9ng/ml’ | ‘≥8ng/ml’ | ‘5-5.9ng/ml’ |
| **…** |  |  |  |  |  |

To address time-varying confounding affected by past tacrolimus exposure, we utilized a Cox marginal structural model (MSM) with Inverse Probability of Treatment Weighting (IPTW). The methods were necessitated by the complex interplay between tacrolimus trough levels and patient outcomes. Tacrolimus levels at each time point are not only influenced by baseline patient characteristics but are also a function of past clinical outcomes and previous tacrolimus exposures. This dynamic relationship necessitates a sophisticated statistical approach that can adequately adjust for these time-varying confounders, ensuring that the estimated effects of tacrolimus levels on allograft outcomes are accurately captured.

MSM with IPTW involves stabilized weights that balance effect of the time varying confounding. The stabilized weight at time t consisted of the product of the treatment weight and censoring weight, and was computed as follows:

$$\mathrm{sw}_{i}(t)=\prod_{k=1}^{t} \frac{pr(A(k)=a_{i}(k) \mid\overline{A}(k-1)=\overline{a_{i}}(k-1),V=v_{i})}{pr(A(k)=a_{i}(k) \mid\overline{A}(k-1)=\overline{a_{i}}(k-1),\overline{L}(k)=\overline{l_{i}}(k),V=v_{i})}$$

$${\mathrm{sw}_{i}(t)}^{c}=\prod_{k=1}^{t} \frac{pr(C(k)=0 \mid\overline{C}(k-1)=0, \overline{A}(k-1)=\overline{a_{i}}(k-1),V=v_{i})}{pr(C(k)=0 \mid\overline{C}(k-1)=0,\overline{A}(k-1)=\overline{a_{i}}(k-1),\overline{L}(k-1)=\overline{l_{i}}(k-1),V=v_{i})}$$

where A(k) denotes the tacrolimus trough level at time k, $\overline{A}(k-1)$ the tacrolimus level history prior to time k, V the baseline covariate, $C(k)$ the censoring state at time k, and $\overline{L}(k)$ the covariate history up to and including time k.

Treatment weights were calculated based on the inverse of each individual’s probability of belonging to one of the seven tacrolimus trough-level categories at each time points, considering both time-dependent and -independent covariates. Similarly, censoring weights were derived from the probability of being censored at each time periods, also considering both the time-dependent and -independent covariates. Covariates included in the stabilized weight calculations were age, sex, duration of prior dialysis, use of non-tacrolimus immunosuppressants (MMF, MPA, steroids, and other immunosuppressants), induction agents, desensitization status, donor characteristics, outcomes prior to the start of the cohort time (rejection, dysfunction, dnDSA), and time-varying serum creatinine levels.

The dataset structured for survival analysis associates each patient with intervals defined from the 2-month post-transplant start date to the end of each subsequent 2-month period, detailing tacrolimus levels, outcomes, and corresponding stabilized weights as shown below.

| **ID** | **Start time** | **End time** | **Tacrolimus**  **trough level** | **Outcome** | **stabilized weight** |
| --- | --- | --- | --- | --- | --- |
| **Patient 1** | 2 mo. start date | 4 mo. end date | 3.0-3.9 ng/ml | 0 | $\mathrm{sw}_{1}(1){{\times\mathrm{sw}}_{1}(1)}^{c}$ |
| **Patient 1** | 4 mo. start date | 6 mo. end date | 7.0-7.9 ng/ml | 0 | $\mathrm{sw}_{1}(2){{\times\mathrm{sw}}_{1}(2)}^{c}$ |
| **Patient 1** | 6 mo. start date | 8 mo. end date | 6.0-6.9 ng/ml | 0 | $\mathrm{sw}_{1}(3){{\times\mathrm{sw}}_{1}(3)}^{c}$ |
| **Patient 1** | 8 mo. start date | 10 mo. end date | 4.0-4.9 ng/ml | 1 | $\mathrm{sw}_{1}(4){{\times\mathrm{sw}}_{1}(4)}^{c}$ |
| **~~Patient 1~~** | ~~10 mo. start date~~ | ~~12 mo. end date~~ | ~~<3.0 ng/ml~~ | ~~0~~ |  |
| **Patient 2** | 2 mo. start date | 4 mo. end date | <3.0 ng/ml | 0 | $\mathrm{sw}_{1}(1){{\times\mathrm{sw}}_{1}(1)}^{c}$ |
| **Patient 2** | 4 mo. start date | 6 mo. end date | 6.0-6.9 ng/ml | 0 | $\mathrm{sw}_{2}(2){{\times\mathrm{sw}}_{2}(2)}^{c}$ |
| **…** |  |  |  |  |  |

Survival analysis was then conducted with these weighted observations to estimate the risks associated with each tacrolimus level category, with results expressed as adjusted hazard ratio and 95% confidence intervals.

**3. Database variables**:

Variables of the Seoul Cohort database are listed below. All laboratory values collected from preoperative 1 year to graft failure, death, or by December 31, 2021, whichever occurred first. Relevant follow-up data till 1 or 6 years post-transplant (as per the cohort definition) was used for the current study.

| Baseline Recipient characteristics | age, sex, bwt, height, cause of ESRD, hypertension, diabetes, previous RRT, previous RRT duration, ABO, Rh, HLA_A1, HLA_A2, HLA_B1, HLA_B2, HLA_DR1, HLA_DR2, HLA_DQ1, HLA_DQ2 |
| --- | --- |
| Baseline donor characteristics | age, sex, bwt, height, hypertension, diabetes, donor type, ABO, Rh, HLA_A1, HLA_A2, HLA_B1, HLA_B2, HLA_DR1, HLA_DR2, HLA_DQ1, HLA_DQ2, HBs Ag, Anti-HBs, HBc IgG, HBc IgM, HBe Ag, HBe Ab, HBV DNA, HCV ab, HIV Ag/Ab, EBV IgM, EBV IgG, CMV Ag, CMV IgM, CMV IgG, HSV IgM, HSV IgG, BKJC_PCR, |
| Transplant characteristics | transplant type (single, dual, en-bloc), previous transplant, ABO incompatibility, anti_A_IgM, anti_A_IgG, peak_anti_A_IgM, peak_anti_A_IgG, anti_B_IgM, anti_B_IgG, peak_anti_B_IgM, peak_anti_B_IgG, DSA, DSA_sp, DSA_MFI, DSA_MFI_peak, CDCXM_B, CDCXM_B_titer, FXM_B, FXM_B_titer, FXM_T, FXM_T_titer, desensitization, desensitization specifics - medication type & dose, number of plasmapheresis, induction medication & dose, cold ischemic time, warm ischemic time, graft weight |
| Follow-up event | serious infection, serious infection_date, serious infection_organism, PJP infection, PJP infection_date, CMV_viremia, CMV_viremia_date, EBV_viremia, EBV_viremia_date, BK_viremia, BK_viremia_date, malignancy_type, malignancy_date, MACE, MACE_date, MACE_type, MACE_fatal, death, death_date |
| Admission event | admission_date, admission_diagnosis code |
| Medication (date, generic name, dosage) | Immunosuppressants (tacrolimus, cyclosporin A, methylprednisolone, prednisolone, deflazacort, sirolimus, everolimus, mizorbine, mycophenolate mofetil, mycophenolate Na, leflunomide, antithymocyte globulin, Immunoglobulin G, methylprednisolon succinate, rituximab, bortezomib, basiliximab), antiviral medications (CMV, HBV, HSV), TMP/SMX, antifungal agents, antidiabetic medication, antihypertensive medication, lipid lowering agents, uric acid lowering agents, vitamin D analogs, bisphosphonate, calcimimetics |
| Laboratory results (date, test name, results) | HBs Ag, Anti-HBs, HBc IgG, HBc IgM, HBe Ag, HBe Ab, HBV DNA, HCV ab, HIV Ag/Ab, EBV IgM, EBV IgG, CMV Ag, CMV IgM, CMV IgG, HSV IgM, HSV IgG, BKJC_PCR, interferon gamma, complete blood count, lymphocyte, monocyte, eosinophil, absolute neutrophil count, blood urea nitrogen, serum creatinine, MDRDeGFR, CKDeGFR, cystatin C, alkaline phosphatase, total bilirubin, alanine aminotransferase, aspartate aminotransferase, glucose, albumin, protein, phosphorus, calcium, uric acid, chloride, potassium, sodium, c-reactive protein, low-density lipoprotein, high-density lipoprotein, triglycerides, total cholesterol, ionized calcium, intact parathyroid hormone, vitamin D, total carbon dioxide, alpha-fetoprotein, protein induced by vitamin k absence or antagonist-ii, hemoglobin A1c, tacrolimus trough level, cyclosporine trough level, sirolimus trough level, random urine protein, random urine creatinine, random urine protein to creatinine ratio, urinalysis (pH, specific gravity, ketone, nitrite, glucose, protein, leukocyte esterase, red blood cell count, white blood cell count, crystals, casts, bacteria), CMV real-time PCR, CMV antigenemia assay, EBV real-time PCR, BK virus real-time PCR, Pneumocystis jirovecii PCR, Pneumocystis jiroveccii IF staining |
| Biopsy results | biopsy_date, Banff diagnostic categories, Banff lesion score |
| HLA antibody test results | PRA_class I, PRA_class II, DSA_class I, DSA_class I titer, DSA_class II, DSA_class II titer |

**Table S1.** Demographic and clinical characteristics of the 6-year analysis cohort (n=4,488)

|  | Total  (n=4,488) | Tacrolimus trough level (ng/mL) | | | | | | | P-value^a^ | SMD |
| --- | --- | --- | --- | --- | --- | --- | --- | --- | --- | --- |
|  |  | <3.0  (n=109) | 3.0–3.9  (n=311) | 4.0–4.9  (n=779) | 5.0–5.9  (n=1,034) | 6.0–6.9  (n=1,011) | 7.0–7.9  (n=630) | ≥8.0  (n=614) |  |  |
| Recipient characteristics |  |  |  |  |  |  |  |  |  |  |
| Age, years, mean±SD | 43.5±13.4 | 45.5±12.9 | 41.4±13.8 | 42.7±13.6 | 43.0±13.7 | 43.8±13.5 | 44.6±12.8 | 44.3±12.6 | 0.0016 | 4.35 |
| Male sex, n(%) | 2,590 (57.7) | 62 (56.9) | 174 (55.9) | 424 (54.4) | 566 (54.7) | 592 (58.6) | 393 (62.4) | 379 (61.7) | 0.0073 | 5.4 |
| BMI, kg/m^2^, mean±SD | 23.0±38.0 | 22.6±4.3 | 22±4.3 | 22.4±7.0 | 22.3±4.8 | 25.2±79.6 | 22.4±3.9 | 22.6±4.5 | 0.6455 | 0.73 |
| Hypertension, n (%) | 2,877 (64.1) | 62 (56.9) | 163 (52.4) | 475 (61) | 650 (62.9) | 712 (70.4) | 446 (70.8) | 369 (60.1) | 0.0000 | 5.32 |
| Diabetes mellitus, n (%) | 773 (17.2) | 12 (11.0) | 32 (10.3) | 102 (13.1) | 141 (13.6) | 175 (17.3) | 131 (20.8) | 180 (29.3) | 0.0000 | 13.69 |
| Primary etiology of ESRD, n (%) |  |  |  |  |  |  |  |  | 0.0000 | 3.69 |
| Diabetes | 779 (17.4) | 17 (15.6) | 44 (14.1) | 111 (14.2) | 152 (14.7) | 157 (15.5) | 123 (19.6) | 175 (28.5) |  |  |
| Hypertension | 444 (9.9) | 11 (10.1) | 30 (9.6) | 85 (10.9) | 92 (8.9) | 110 (10.9) | 73 (11.6) | 43 (7.0) |  |  |
| GN | 865 (19.3) | 29 (26.6) | 74 (23.8) | 173 (22.2) | 213 (20.6) | 180 (17.8) | 96 (15.3) | 100 (16.3) |  |  |
| PKD | 190 (4.2) | 5 (4.6) | 9 (2.9) | 26 (3.3) | 50 (4.8) | 43 (4.3) | 32 (5.1) | 25 (4.1) |  |  |
| IgA nephropathy | 447 (10.0) | 7 (6.4) | 35 (11.3) | 72 (9.2) | 110 (10.6) | 108 (10.7) | 69 (11.0) | 46 (7.5) |  |  |
| Others | 510 (11.4) | 9 (8.3) | 44 (14.1) | 94 (12.1) | 131 (12.7) | 121 (12.0) | 59 (9.4) | 52 (8.5) |  |  |
| Unknown | 1,251 (27.9) | 31 (28.4) | 75 (24.1) | 218 (28.0) | 285 (27.6) | 292 (28.9) | 177 (28.1) | 173 (28.2) |  |  |
| Repeat transplant, n (%) | 406 (9.0) | 7 (6.4) | 27 (8.7) | 67 (8.6) | 81 (7.8) | 99 (9.8) | 63 (10.0) | 62 (10.1) | 0.5150 | 2.13 |
| Pre-transplant dialysis, n (%) | 3,775 (84.1) | 92 (84.4) | 250 (80.4) | 647 (83.1) | 864 (83.6) | 862 (85.3) | 529 (84.0) | 531 (86.5) | 0.2595 | 2.94 |
| Time on dialysis, mo., mean±SD | 209.3±442.1 | 333.9±553.0 | 337.6±554.3 | 259±491.2 | 168.3±389.8 | 152.4±371.2 | 195.1±427.8 | 236.7±470.3 | 0.0000 | 5.63 |
| Transplant characteristics |  |  |  |  |  |  |  |  |  |  |
| PRA, %, mean±SD |  |  |  |  |  |  |  |  |  |  |
| Class I | 10.6±25.1 | 13.1±29.8 | 10.4±25.7 | 10.6±25.6 | 10.7±25.1 | 12.4±26.8 | 7.8±20.5 | 9.8±24.4 | 0.2937 | 1.32 |
| Class II | 10.0±24.4 | 15.3±32 | 11.1±27.2 | 10.2±23.9 | 8.4±22.6 | 10.4±25.0 | 8.3±21.4 | 13±27.1 | 0.0978 | 0.03 |
| HLA-A/B/DR antigen mismatches, mean±SD | 3.4±1.5 | 3.5±1.6 | 3.3±1.5 | 3.5±1.4 | 3.3±1.5 | 3.3±1.5 | 3.4±1.4 | 3.4±1.4 | 0.0399 | 0.72 |
| Pre-DSA positivity, n (%) | 227 (9.1) | 7 (9.9) | 16 (9.1) | 42 (10.7) | 39 (7.8) | 51 (8.9) | 40 (10.7) | 32 (8.0) | 0.6857 | 0.89 |
| Type of DSA, n (%) |  |  |  |  |  |  |  |  | 0.1198 | 8.91 |
| Non-HLA DSA | 2 (0.9) | 0 | 0 | 0 | 1 (2.6) | 0 | 0 | 1 (3.1) |  |  |
| HLA I DSA | 94 (41.4) | 4 (57.1) | 5 (31.3) | 17 (40.5) | 21 (53.8) | 19 (37.3) | 18 (45) | 10 (31.3) |  |  |
| HLA II DSA | 87 (38.3) | 3 (42.9) | 9 (56.3) | 19 (45.2) | 7 (17.9) | 24 (47.1) | 16 (40.0) | 9 (28.1) |  |  |
| HLA I and II DSA | 44 (19.4) |  | 2 (12.5) | 6 (14.3) | 10 (25.6) | 8 (15.7) | 6 (15.0) | 12 (37.5) |  |  |
| Desensitization, n (%) | 800 (17.8) | 26 (23.9) | 70 (22.5) | 158 (20.3) | 182 (17.6) | 175 (17.3) | 96 (15.2) | 93 (15.1) | 0.0106 | 5.74 |
| ABO incompatible, n (%) | 375 (9.9) | 8 (10.1) | 32 (14.6) | 62 (10.0) | 91 (10.0) | 86 (9.5) | 50 (9.2) | 46 (9.0) | 0.3840 | 1.22 |
| Crossmatch positivity, n (%) | 217 (4.8) | 3 (2.8) | 18 (5.8) | 50 (6.4) | 49 (4.7) | 47 (4.7) | 25 (4.0) | 25 (4.1) | 0.2552 | 10.25 |
| Induction therapy, n (%) |  |  |  |  |  |  |  |  | 0.0000 | 4.92 |
| None | 246 (5.5) | 7 (6.4) | 9 (2.9) | 23 (3.0) | 58 (5.6) | 66 (6.5) | 43 (6.8) | 40 (6.5) |  |  |
| Basiliximab | 3,684 (82.1) | 85 (78.0) | 280 (90.0) | 699 (89.7) | 877 (84.8) | 811 (80.2) | 486 (77.1) | 446 (72.6) |  |  |
| ATG | 549 (12.2) | 17 (15.6) | 22 (7.1) | 55 (7.1) | 97 (9.4) | 130 (12.9) | 101 (16.0) | 127 (20.7) |  |  |
| Others | 9 (0.2) |  |  | 2 (0.3) | 2 (0.2) | 4 (0.4) |  | 1 (0.2) |  |  |
| IS at 1-year post-transplant, n (%)^b^ |  |  |  |  |  |  |  |  |  |  |
| Tacrolimus | 4,068 (90.6) | 104 (95.4) | 295 (94.9) | 721 (92.6) | 942 (91.1) | 893 (88.3) | 556 (88.3) | 557 (90.7) | 0.0006 | 1.95 |
| Once daily | 108 (2.4) | 6 (5.5) | 28 (9) | 39 (5) | 16 (1.5) | 13 (1.3) | 5 (0.8) | 1 (0.2) | 0.0000 | 13.04 |
| Twice daily | 3,961 (88.3) | 98 (89.9) | 267 (85.9) | 682 (87.5) | 926 (89.6) | 881 (87.1) | 551 (87.5) | 556 (90.6) | 0.1848 | 3.62 |
| Cyclosporine | 12 (0.3) | 0 | 2 (0.6) | 1 (0.1) | 5 (0.5) | 3 (0.3) | 1 (0.2) | 0 | 0.3933 | 3.84 |
| MMF or EC-MPA | 2,445 (54.5) | 63 (57.8) | 193 (62.1) | 481 (61.7) | 597 (57.7) | 503 (49.8) | 283 (44.9) | 325 (52.9) | 0.0000 | 7.81 |
| Steroid | 2,055 (45.8) | 57 (52.3) | 174 (55.9) | 387 (49.7) | 527 (51) | 431 (42.6) | 231 (36.7) | 248 (40.4) | 0.0000 | 9.81 |
| Others | 135 (3.0) | 4 (3.7) | 12 (3.9) | 26 (3.3) | 29 (2.8) | 27 (2.7) | 20 (3.2) | 17 (2.8) | 0.9246 | 1.84 |
| Donor information |  |  |  |  |  |  |  |  |  |  |
| Age, years, mean±SD | 42.4±12.8 | 45.5±11.7 | 42.8±12.9 | 43±12.7 | 42.7±13.0 | 42.3±12.5 | 41.7±12.3 | 41.2±13.6 | 0.0163 | 5.62 |
| Male sex, n (%) | 2,420 (53.9) | 53 (48.6) | 155 (49.8) | 401 (51.5) | 566 (54.7) | 561 (55.5) | 346 (54.9) | 338 (55.0) | 0.3174 | 3.34 |
| BMI, kg/m^2^, mean±SD | 23.8±11.5 | 23.3±3.0 | 23.6±3.5 | 23.2±3.1 | 23.9±11.3 | 24.2±14.8 | 24.6±18.4 | 23.5±3.3 | 0.4441 | 2.02 |
| Hypertension, n (%) | 275 (9.7) | 4 (5.0) | 18 (8.0) | 46 (9.6) | 63 (11.1) | 53 (8.7) | 45 (10.5) | 46 (10.6) | 0.4846 | 1.24 |
| Donor type, n (%) |  |  |  |  |  |  |  |  | 0.0873 | 1.85 |
| Living related | 2,179 (48.6) | 42 (38.5) | 151 (48.6) | 371 (47.6) | 508 (49.1) | 509 (50.3) | 321 (51.0) | 277 (45.1) |  |  |
| Living non-related | 800 (17.8) | 30 (27.5) | 68 (21.9) | 138 (17.7) | 170 (16.4) | 179 (17.7) | 106 (16.8) | 109 (17.8) |  |  |
| Deceased | 1509 (33.6) | 37 (33.9) | 92 (29.6) | 270 (34.6) | 356 (34.4) | 323 (32) | 203 (32.3) | 228 (37.1) |  |  |

ATG, anti-thymocyte globulin; BMI, body mass index; DSA, donor-specific antibody; EC-MPA, enteric coated mycophenolic acid; ESRD, end-stage renal disease; GN, glomerulonephritis; HLA, human leukocyte antigen; IgA, immunoglobulin A; IS, immunosuppressant; MMF, mycophenolate mofetil; PKD, polycystic kidney disease; PRA, panel reactive antibodies; SD, standard deviation; SMD, standardized mean difference

^a^P-value calculated by $\chi^{2}$ test (categorical covariate) and t-test (continuous covariate).

^b^Each medication prescription data were retrieved separately from the pre-defined period of post-transplant 12±1 months.

**Table S2.** Periodic mean tacrolimus trough levels 2–12 and 12–72 months post-transplant

|  | Tacrolimus trough level range (ng/mL) | | | | | | |
| --- | --- | --- | --- | --- | --- | --- | --- |
|  | <3.0 | 3.0–3.9 | 4.0–4.9 | 5.0–5.9 | 6.0–6.9 | 7.0–7.9 | ≥8.0 |
| ***Number and percent of patients with average 2-month tacrolimus trough level for every 2 months from post-transplant 2–12 months, n (%)*** | | | | | | | |
| 2–4 mo. | 154 (1.5) | 298 (2.9) | 707 (6.8) | 1222 (11.8) | 1713 (16.6) | 2106 (20.4) | 4129 (40.0) |
| 4–6 mo. | 195 (1.9) | 434 (4.3) | 901 (8.9) | 1568 (15.5) | 1896 (18.7) | 1947 (19.2) | 3183 (31.4) |
| 6–8 mo. | 220 (2.2) | 453 (4.6) | 1053 (10.6) | 1628 (16.4) | 2074 (20.9) | 1808 (18.2) | 2675 (27.0) |
| 8–10 mo. | 262 (2.7) | 487 (5.0) | 1132 (11.6) | 1790 (18.4) | 2027 (20.8) | 1664 (17.1) | 2373 (24.4) |
| 10–12 mo. | 260 (2.7) | 543 (5.7) | 1226 (12.9) | 1828 (19.2) | 2037 (21.4) | 1628 (17.1) | 1981 (20.9) |
| ***Number and percent of patients with average 1-year tacrolimus trough level for every 12 months from post-transplant 12–72 months, n (%)*** | | | | | | | |
| 12–24 mo. | 109 (2.4) | 311 (6.9) | 779 (17.4) | 1034 (23.0) | 1011 (22.5) | 630 (14.0) | 614 (13.7) |
| 24–36 mo. | 175 (4.1) | 418 (9.7) | 895 (20.7) | 1091 (25.2) | 843 (19.5) | 469 (10.9) | 431 (10.0) |
| 36–48 mo. | 207 (4.9) | 473 (11.3) | 898 (21.4) | 1025 (24.5) | 774 (18.5) | 476 (11.4) | 340 (8.1) |
| 48–60 mo. | 189 (4.6) | 465 (11.4) | 934 (22.8) | 967 (23.6) | 752 (18.4) | 449 (11.0) | 340 (8.3) |
| 60–72 mo. | 161 (4.1) | 499 (12.6) | 872 (22.1) | 989 (25.0) | 693 (17.5) | 429 (10.9) | 307 (7.8) |

Mo, months

**Table S3.** Crude incidence of primary and secondary transplant outcomes by baseline tacrolimus trough level

| **1-year transplant outcome** |  |  |  |  |  |  |  |  |  |
| --- | --- | --- | --- | --- | --- | --- | --- | --- | --- |
|  | Total  (n=10,329) | Tacrolimus trough level at 2 months (ng/mL) | | | | | | | p-value |
|  |  | <3.0  (n=154) | 3.0–3.9  (n=298) | 4.0–4.9  (n=707) | 5.0–5.9  (n=1,222) | 6.0–6.9  (n=1,713) | 7.0–7.9  (n=2,106) | ≥8  (n=4,129) |  |
| **Primary objective** |  |  |  |  |  |  |  |  |  |
| Composite allograft outcome, n (%) | 1,161 (11.2) | 32 (22.7) | 46 (16.7) | 94 (14.6) | 134 (12) | 177 (11.1) | 254 (13.1) | 424 (11.2) | <0.0001 |
| Biopsy-proven rejection | 911 (8.8) | 24 (15.6) | 28 (9.4) | 54 (7.6) | 87 (7.1) | 137 (8) | 211 (10) | 370 (9) | 0.0030 |
| Kidney dysfunction | 471 (4.6) | 25 (16.2) | 21 (7) | 47 (6.6) | 76 (6.2) | 71 (4.1) | 92 (4.4) | 139 (3.4) | <0.0001 |
| dnDSA development | 121 (1.2) | 2 (1.4) | 10 (3.6) | 28 (4.3) | 19 (1.7) | 21 (1.3) | 19 (1) | 22 (0.6) | <0.0001 |
| Death-censored graft failure | 110 (1.1) | 10 (6.5) | 8 (2.7) | 8 (1.1) | 11 (0.9) | 20 (1.2) | 22 (1) | 31 (0.8) | <0.0001 |
| **Secondary objective** |  |  |  |  |  |  |  |  |  |
| Severe infection, n (%) | 847 (8.2) | 24 (17.9) | 33 (12.1) | 75 (11.3) | 112 (9.6) | 134 (8.1) | 148 (7.4) | 321 (8) | <0.0001 |
| Cardiovascular event, n (%) | 15 (0.1) | 0 (0.0) | 0 (0.0) | 1 (0.1) | 4 (0.3) | 6 (0.4) | 1 (0) | 3 (0.1) | 0.0785 |
| Patient mortality, n (%) | 81 (0.8) | 3 (1.9) | 4 (1.3) | 4 (0.6) | 7 (0.6) | 12 (0.7) | 16 (0.8) | 35 (0.8) | 0.4944 |
| **2–6-year transplant outcome** |  |  |  |  |  |  |  |  |  |
|  | Total  (n=4,488) | Tacrolimus trough level at 1 year (ng/mL) | | | | | | | p-value |
|  |  | <3.0  (n=109) | 3.0–3.9  (n=311) | 4.0–4.9  (n=707) | 5.0–5.9  (n=1,034) | 6.0–6.9  (n=1,011) | 7.0–7.9  (n=630) | ≥8.0  (n=614) |  |
| **Primary objective** |  |  |  |  |  |  |  |  |  |
| Composite allograft outcome, n(%) | 1,037 (23.1) | 36 (35.0) | 82 (28.0) | 195 (26.5) | 254 (26.0) | 202 (21.2) | 132 (22.0) | 136 (23.1) | 0.0036 |
| Biopsy-proven rejection | 731 (16.3) | 19 (17.4) | 51 (16.4) | 143 (18.4) | 186 (18) | 157 (15.5) | 94 (14.9) | 81 (13.2) | 0.1106 |
| Kidney dysfunction | 438 (9.8) | 18 (16.5) | 36 (11.6) | 75 (9.6) | 106 (10.3) | 83 (8.2) | 58 (9.2) | 62 (10.1) | 0.1178 |
| dnDSA development | 237 (5.3) | 10 (9.7) | 25 (8.5) | 57 (7.7) | 45 (4.6) | 38 (4.0) | 35 (5.8) | 27 (4.6) | 0.0010 |
| Death-censored graft failure | 266 (5.9) | 12 (11.4) | 33 (10.6) | 45 (5.8) | 73 (7.1) | 42 (4.2) | 26 (4.1) | 35 (5.7) | <0.0001 |
| **Secondary objective** |  |  |  |  |  |  |  |  |  |
| Severe infection, n (%) | 526 (11.7) | 8 (9.4) | 43 (15.6) | 89 (12.6) | 111 (11.8) | 117 (13.0) | 73 (13.1) | 85 (15.8) | 0.2812 |
| Cardiovascular event, n (%) | 25 (0.6) | 1 (1.0) | 3 (1.0) | 3 (0.4) | 6 (0.6) | 6 (0.6) | 3 (0.5) | 3 (0.5) | 0.9353 |
| Malignancy, n (%) | 157 (3.5) | 3 (2.9) | 10 (3.3) | 29 (3.8) | 31 (3.0) | 35 (3.5) | 22 (3.5) | 27 (4.4) | 0.8715 |
| Patient mortality, n (%) | 129 (2.9) | 6 (5.5) | 10 (3.2) | 14 (1.8) | 29 (2.8) | 31 (3.1) | 18 (2.9) | 21 (3.4) | 0.3322 |

dnDSA, de novo donor-specific antibodies

**Table S4.** Relative hazards of biopsy-proven rejection, renal dysfunction, de novo donor-specific antibodies, and death-censored graft failure 2–12 months post-transplant according to periodic mean tacrolimus trough range

| Tacrolimus trough level (ng/mL) | Composite allograft outcome | | | Biopsy proven rejection | | Kidney dysfunction (eGFR<30mL/min/1.73m^2^) | | Development of de novo DSA | | Death-censored graft failure | |
| --- | --- | --- | --- | --- | --- | --- | --- | --- | --- | --- | --- |
|  | HR (95% CI) | p-value | | HR (95% CI) | p-value | HR (95% CI) | p-value | HR (95% CI) | p-value | HR (95% CI) | p-value |
| **Unadjusted analysis** |  | |  |  |  |  |  |  |  |  |  |
| <3 | 4.70 (3.98,5.55) | <0.0001 | | 2.73 (2.21,3.37) | <0.0001 | 7.42 (5.77,9.55) | <0.0001 | 14.93 (8.22,27.13) | <0.0001 | 13.73 (7.59,24.82) | <0.0001 |
| 3–3.9 | 1.33 (1.00,1.77) | 0.0486 | | 1.23 (0.90,1.69) | 0.1997 | 1.74 (1.14,2.65) | 0.0099 | 6.27 (2.87,13.68) | <0.0001 | 4.86 (2.23,10.58) | 0.0001 |
| 4–4.9 | 1.02 (0.82,1.28) | 0.8497 | | 0.81 (0.62,1.06) | 0.1253 | 1.07 (0.74,1.55) | 0.7114 | 4.89 (2.52,9.50) | <0.0001 | 2.49 (1.19,5.22) | 0.0156 |
| 5–5.9 | 0.67 (0.54,0.84) | 0.0003 | | 0.69 (0.55,0.87) | 0.0018 | 0.71 (0.50,1.02) | 0.0677 | 1.28 (0.57,2.85) | 0.5448 | 1.42 (0.67,3.02) | 0.3649 |
| 6–6.9 | 0.79 (0.65,0.95) | 0.013 | | 0.83 (0.68,1.02) | 0.0713 | 0.9 (0.65,1.23) | 0.4978 | 1.69 (0.84,3.39) | 0.1398 | 0.86 (0.38,1.95) | 0.7151 |
| 7–7.9 | 0.71 (0.58,0.86) | 0.0006 | | 0.87 (0.71,1.06) | 0.1592 | 0.59 (0.4,0.85) | 0.0044 | 0.7 (0.28,1.71) | 0.4285 | 0.81 (0.34,1.90) | 0.6244 |
| ≥8 | 1 |  | | 1 |  | 1 |  | 1 |  | 1 |  |
| **Adjusted analysis** |  |  | |  |  |  |  |  |  |  |  |
| <3 | 4.74 (4,5.63) | <0.0001 | | 2.97 (2.39,3.69) | <0.0001 | 6.85 (5.29,8.86) | <0.0001 | 14.32 (7.77,26.38) | 0.0001 | 12.42 (6.77,22.79) | <0.0001 |
| 3–3.9 | 1.40 (1.05,1.87) | 0.0229 | | 1.32 (0.95,1.81) | 0.0934 | 1.55 (1.02,2.38) | 0.0424 | 5.47 (2.48,12.09) | 0.0001 | 4.80 (2.18,10.57) | 0.0001 |
| 4–4.9 | 1.02 (0.82,1.29) | 0.834 | | 0.83 (0.64,1.09) | 0.1773 | 1.02 (0.70,1.49) | 0.9038 | 4.31 (2.20,8.46) | <0.0001 | 2.50 (1.18,5.29) | 0.0165 |
| 5–5.9 | 0.69 (0.55,0.85) | 0.0007 | | 0.71 (0.56,0.90) | 0.0041 | 0.68 (0.47,0.98) | 0.0401 | 1.19 (0.53,2.67) | 0.6698 | 1.41 (0.66,3.03) | 0.3714 |
| 6–6.9 | 0.81 (0.67,0.98) | 0.0327 | | 0.85 (0.69,1.04) | 0.1164 | 0.87 (0.63,1.19) | 0.3715 | 1.62 (0.81,3.26) | 0.175 | 0.87 (0.38,1.98) | 0.7373 |
| 7–7.9 | 0.73 (0.6,0.89) | 0.0016 | | 0.88 (0.72,1.08) | 0.2252 | 0.58 (0.4,0.84) | 0.004 | 0.69 (0.28,1.69) | 0.4141 | 0.83 (0.35,1.96) | 0.6741 |
| ≥8 | 1 |  | | 1 |  | 1 |  | 1 |  | 1 |  |

CI, confidence interval; dnDSA, de novo donor-specific antibodies; HR, hazard ratio; IPTW, inverse probability of treatment weighting

P-value was calculated by time-varying Cox proportional hazard analysis with periodic mean tacrolimus trough concentration as the time-varying exposure variable (unadjusted analysis). The adjusted analysis incorporated the IPTW method with stabilized weight, which included: sex; age; previous dialysis months; use of immunosuppressants other than tacrolimus; use of induction agents; desensitization; donor age; donor sex; and serum creatinine (time-dependent covariate) for the calculation of stabilized weights.

**Table S5.** Relative hazards of the 1-year composite allograft outcome according to periodic mean tacrolimus trough range in pre-defined subgroups

|  | Tacrolimus trough level groups (ng/mL) | | | | | | |
| --- | --- | --- | --- | --- | --- | --- | --- |
|  | <3.0 | 3.0–3.9 | 4.0–4.9 | 5.0–5.9 | 6.0–6.9 | 7.0–7.9 | ≥8.0 (Ref) |
|  | aHR (95% CI) | aHR (95% CI) | aHR (95% CI) | aHR (95% CI) | aHR (95% CI) | aHR (95% CI) | aHR (95% CI) |
| All subjects | 4.74 (4.00,5.63)*** | 1.4 (1.05,1.87)* | 1.02 (0.82,1.29) | 0.69 (0.55,0.85)*** | 0.81 (0.67,0.98)* | 0.73 (0.60,0.89)** | 1 |
| Age (years) |  |  |  |  |  |  |  |
| <18 | 0.91 (0.21,3.85) | 0.78 (0.16,3.71) | 0.43 (0.12,1.58) | 0.33 (0.12,0.93)* | 0.87 (0.42,1.80) | 0.52 (0.20,1.32) | 1 |
| 19–64 | 5.14 (4.21,6.26)*** | 1.67 (1.22,2.28)** | 1.07 (0.82,1.38) | 0.74 (0.58,0.95)* | 0.84 (0.67,1.04) | 0.74 (0.59,0.93)* | 1 |
| ≥65 | 4.78 (3.23,7.07)*** | 0.63 (0.25,1.59) | 1.06 (0.63,1.77) | 0.59 (0.34,1.00) | 0.65 (0.41,1.03) | 0.72 (0.46,1.12) | 1 |
| Diabetes |  |  |  |  |  |  |  |
| Yes | 6.04 (4.17,8.73)*** | 1.06 (0.42,2.68) | 1.55 (0.91,2.62) | 0.79 (0.48,1.30) | 0.94 (0.63,1.41) | 0.98 (0.66,1.44) | 1 |
| No | 4.57 (3.76,5.57)*** | 1.45 (1.06,1.97)** | 0.97 (0.75,1.25) | 0.67 (0.52,0.85)** | 0.78 (0.63,0.97)* | 0.66 (0.52,0.83)*** | 1 |
| Hypertension |  |  |  |  |  |  |  |
| Yes | 5.23 (4.19,6.52)*** | 1.74 (1.18,2.57)** | 1.10 (0.80,1.50) | 0.84 (0.64,1.10) | 0.88 (0.69,1.12) | 0.85 (0.67,1.07) | 1 |
| No | 16.90 (12.80,22.40)*** | 1.02 (0.57,1.83) | 0.91 (0.59,1.41) | 0.61 (0.40,0.95)* | 0.76 (0.51,1.12) | 0.65 (0.42,1.00) | 1 |
| Donor type |  |  |  |  |  |  |  |
| Living | 6.48 (5.00,8.38)*** | 1.24 (0.73,2.09) | 1.05 (0.72,1.52) | 0.75 (0.53,1.04) | 0.75 (0.55,1.02) | 0.74 (0.54,1.00) | 1 |
| Deceased | 4.07 (3.22,5.14)*** | 1.58 (1.11,2.24)* | 1.04 (0.78,1.40) | 0.66 (0.50,0.88)** | 0.83 (0.65,1.06) | 0.74 (0.57,0.95)* | 1 |
| Desensitization |  |  |  |  |  |  |  |
| Yes | 3.18 (2.16,4.69)*** | 1.42 (0.80,2.51) | 0.73 (0.44,1.21) | 0.68 (0.43,1.08) | 0.65 (0.43,0.98)* | 0.68 (0.45,1.03) | 1 |
| No | 5.43 (4.48,6.58)*** | 1.41 (1.01,1.98)* | 1.12 (0.87,1.44) | 0.69 (0.54,0.88)** | 0.86 (0.69,1.06) | 0.73 (0.58,0.91)** | 1 |
| Previous BPR^a^ |  |  |  |  |  |  |  |
| Yes | 5.00 (3.42,7.30)*** | 0.96 (0.45,2.01) | 1.08 (0.68,1.71) | 0.85 (0.57,1.29) | 0.74 (0.50,1.09) | 0.88 (0.61,1.28) | 1 |
| No | 5.03 (4.13,6.12)*** | 1.52 (1.11,2.09)** | 1.03 (0.79,1.34) | 0.64 (0.49,0.83)*** | 0.85 (0.68,1.05) | 0.68 (0.54,0.86)** | 1 |

aHR, adjusted hazard ratio; BPR, biopsy-proven rejection; CI, confidence interval

^a^ Previous BPR defined as history of biopsy-proven rejection within 2 months post-transplant.

 *, **, and *** indicate HRs with p-values <0.05, 0.01, or 0.001, respectively.

**Table S6.** Relative hazards of biopsy-proven rejection, kidney dysfunction, de novo donor-specific antibodies, death-censored graft failure 12–72 months post-transplant according to periodic mean tacrolimus trough range

| Tacrolimus trough level (ng/mL) | Composite allograft outcome | | Biopsy-proven rejection | | Kidney dysfunction (eGFR<30mL/min/1.73m^2^) | | Development of  de novo DSA | | Death-censored graft failure | |
| --- | --- | --- | --- | --- | --- | --- | --- | --- | --- | --- |
|  | HR (95% CI) | p-value | HR (95% CI) | p-value | HR (95% CI) | p-value | HR (95% CI) | p-value | HR (95% CI) | p-value |
| **Unadjusted analysis** |  |  |  |  |  |  |  |  |  |  |
| <3.0 | 3.23 (2.56,4.06) | <0.0001 | 2.97 (2.19,4.01) | 0.0001 | 2.77 (2.00,3.83) | <0.0001 | 2.58 (1.48,4.51) | 0.0008 | 3.33 (2.13,5.22) | 0.0001 |
| 3.0–3.9 | 0.86 (0.64,1.14) | 0.2892 | 1.16 (0.82,1.64) | 0.4100 | 0.67 (0.44,1.02) | 0.0636 | 1.89 (1.02,3.50) | 0.0422 | 1.10 (0.65,1.85) | 0.7289 |
| 4.0–4.9 | 0.94 (0.74,1.19) | 0.5890 | 1.10 (0.81,1.50) | 0.5544 | 0.60 (0.41,0.86) | 0.0056 | 1.66 (0.95,2.90) | 0.0774 | 0.85 (0.53,1.37) | 0.5011 |
| 5.0–5.9 | 0.72 (0.57,0.92) | 0.0094 | 0.97 (0.71,1.32) | 0.8290 | 0.49 (0.34,0.70) | 0.0001 | 1.07 (0.60,1.90) | 0.8159 | 0.54 (0.33,0.89) | 0.0166 |
| 6.0–6.9 | 0.69 (0.53,0.89) | 0.0044 | 0.99 (0.72,1.36) | 0.9400 | 0.52 (0.35,0.76) | 0.0008 | 0.79 (0.42,1.46) | 0.4494 | 0.50 (0.29,0.86) | 0.0120 |
| 7.0–7.9 | 0.82 (0.62,1.09) | 0.1687 | 1.11 (0.79,1.57) | 0.5383 | 0.56 (0.37,0.87) | 0.0091 | 0.66 (0.32,1.35) | 0.2569 | 0.71 (0.40,1.24) | 0.2282 |
| ≥8.0 | 1 |  | 1 |  | 1 |  | 1 |  | 1 |  |
| **Adjusted analysis** |  |  |  |  |  |  |  |  |  |  |
| <3.0 | 2.94 (2.33,3.71) | <0.0001 | 2.66 (1.96,3.61) | <0.0001 | 2.46 (1.77,3.41) | <0.0001 | 2.67 (1.52,4.67) | 0.0006 | 3.27 (2.08,5.13) | <0.0001 |
| 3.0–3.9 | 0.86 (0.64,1.15) | 0.3106 | 1.11 (0.78,1.58) | 0.5629 | 0.65 (0.42,1.00) | 0.0494 | 1.85 (1.00,3.44) | 0.0511 | 1.20 (0.71,2.04) | 0.4883 |
| 4.0–4.9 | 0.91 (0.71,1.16) | 0.4451 | 1.04 (0.76,1.43) | 0.7899 | 0.56 (0.39,0.81) | 0.0020 | 1.61 (0.92,2.84) | 0.0975 | 0.93 (0.57,1.50) | 0.7681 |
| 5.0–5.9 | 0.68 (0.53,0.87) | 0.0024 | 0.88 (0.64,1.20) | 0.4197 | 0.42 (0.29,0.61) | <0.0001 | 1.10 (0.62,1.96) | 0.7473 | 0.58 (0.35,0.97) | 0.0375 |
| 6.0–6.9 | 0.65 (0.50,0.85) | 0.0012 | 0.92 (0.67,1.27) | 0.6038 | 0.46 (0.31,0.67) | 0.0001 | 0.81 (0.43,1.50) | 0.4941 | 0.53 (0.31,0.91) | 0.0218 |
| 7.0–7.9 | 0.81 (0.61,1.06) | 0.1255 | 1.08 (0.77,1.53) | 0.6502 | 0.52 (0.34,0.79) | 0.0025 | 0.67 (0.32,1.37) | 0.2677 | 0.72 (0.41,1.27) | 0.2544 |
| ≥8.0 | 1 |  | 1 |  | 1 |  | 1 |  | 1 |  |

CI, confidence interval; DSA, donor-specific antibodies; eGFR, estimated glomerular filtration; HR, hazard ratio; IPTW, inverse probability of treatment weighting

P-value was calculated by time-varying Cox proportional hazard analysis using periodic mean tacrolimus trough concentrations as the time-varying exposure variable (unadjusted analysis). The adjusted analysis incorporated the IPTW method with stabilized weight, which included: sex; age; previous dialysis months; use of immunosuppressant other than tacrolimus; use of induction agents; desensitization; donor age; donor sex; and serum creatinine (time-dependent covariate) for the calculation of stabilized weights.

**Table S7.** Relative hazards of safety outcomes of severe infection, cardiovascular events, and patient mortality 2–12 months post-transplant according to periodic mean tacrolimus trough range

| Tacrolimus trough level (ng/mL) | Severe infection | | Cardiovascular event | | Patient mortality | |
| --- | --- | --- | --- | --- | --- | --- |
|  | HR (95% CI) | p-value^a^ | HR (95% CI) | p-value^a^ | HR (95% CI) | p-value^a^ |
| **Unadjusted analysis** |  |  |  |  |  |  |
| <3.0 | 5.51 (4.55,6.67) | <0.0001 | 8.51 (2.02,35.85) | 0.0035 | 6.00 (3.38,10.65) | <0.0001 |
| 3.0–3.9 | 1.05 (0.72,1.53) | 0.7916 | –^b^ | – | 0.55 (0.13,2.34) | 0.4188 |
| 4.0–4.9 | 0.92 (0.69,1.21) | 0.5407 | 2.9 (0.58,14.44) | 0.1946 | 1.14 (0.52,2.47) | 0.7491 |
| 5.0–5.9 | 0.92 (0.73,1.16) | 0.4672 | 0.58 (0.06,5.64) | 0.6417 | 0.32 (0.11,0.92) | 0.0345 |
| 6.0–6.9 | 0.74 (0.59,0.94) | 0.0134 | 0.94 (0.16,5.68) | 0.9505 | 0.66 (0.31,1.38) | 0.2693 |
| 7.0–7.9 | 0.77 (0.61,0.97) | 0.0289 | 0.48 (0.05,4.66) | 0.5296 | 0.49 (0.21,1.14) | 0.0966 |
| ≥8.0 | 1 |  | 1 |  | 1 |  |
| **Adjusted analysis** |  |  |  |  |  |  |
| <3.0 | 5.49 (4.52,6.68) | <0.0001 | 4.78 (1.07,21.29) | 0.0401 | 5.78 (3.19,10.48) | <0.0001 |
| 3.0–3.9 | 1.09 (0.75,1.59) | 0.6494 | – | – | 0.55 (0.13,2.37) | 0.4244 |
| 4.0–4.9 | 0.96 (0.72,1.27) | 0.7577 | 1.83 (0.35,9.52) | 0.4704 | 1.20 (0.55,2.64) | 0.6488 |
| 5.0–5.9 | 0.94 (0.74,1.19) | 0.6121 | 0.30 (0.03,3.00) | 0.3058 | 0.32 (0.11,0.94) | 0.0377 |
| 6.0–6.9 | 0.79 (0.62,1.00) | 0.0501 | 0.69 (0.11,4.27) | 0.6932 | 0.69 (0.32,1.45) | 0.3264 |
| 7.0–7.9 | 0.81 (0.64,1.03) | 0.0857 | 0.40 (0.04,3.98) | 0.4363 | 0.51 (0.22,1.19) | 0.1178 |
| ≥8.0 | 1 |  | 1 |  | 1 |  |

CI, confidence interval; HR, hazard ratio; IPTW, inverse probability of treatment weighting

^a^P-value was calculated by time-varying Cox proportional hazard analysis with periodic mean tacrolimus trough concentration as the time-varying exposure variable (unadjusted analysis). The adjusted analysis incorporated the IPTW method with stabilized weight, which included: sex; age; previous dialysis months; use of immunosuppressants other than tacrolimus; use of induction agents; desensitization; donor age; donor sex; and serum creatinine (time-dependent covariate) for the calculation of stabilized weights.

^b^Hazard ratios for cardiovascular event were not estimable due to small number of outcome event.

**Table S8.** Relative hazards of safety outcomes of severe infection, cardiovascular events, and patient mortality 12–72 months post-transplant according to yearly mean tacrolimus trough range

| Tacrolimus trough level (ng/mL) | Severe infection | | Cardiovascular event | | Patient mortality | | Malignancy | |
| --- | --- | --- | --- | --- | --- | --- | --- | --- |
|  | HR (95% CI) | p-value^a^ | HR (95% CI) | p-value^a^ | HR (95% CI) | p-value^a^ | HR (95% CI) | p-value^a^ |
| **Unadjusted analysis** |  |  |  |  |  |  |  |  |
| <3.0 | 0.84 (0.60,1.16) | 0.2868 | 1.30 (0.31,5.44) | 0.7208 | 2.01 (1.14,3.54) | 0.0153 | 0.92 (0.48,1.78) | 0.8073 |
| 3.0–3.9 | 0.57 (0.40,0.81) | 0.0017 | 0.86 (0.17,4.27) | 0.8534 | 0.52 (0.24,1.12) | 0.0964 | 0.80 (0.41,1.57) | 0.5108 |
| 4.0–4.9 | 0.47 (0.34,0.63) | <0.0001 | 0.43 (0.09,2.13) | 0.3016 | 0.38 (0.19,0.74) | 0.0049 | 0.65 (0.36,1.19) | 0.1647 |
| 5.0–5.9 | 0.39 (0.29,0.52) | <0.0001 | 0.61 (0.15,2.55) | 0.4963 | 0.36 (0.19,0.69) | 0.0023 | 0.83 (0.48,1.46) | 0.5217 |
| 6.0–6.9 | 0.50 (0.37,0.68) | <0.0001 | 0.47 (0.09,2.31) | 0.3503 | 0.53 (0.28,0.99) | 0.0460 | 0.75 (0.41,1.35) | 0.3366 |
| 7.0–7.9 | 0.70 (0.51,0.96) | 0.0259 | 0.76 (0.15,3.76) | 0.7337 | 0.57 (0.28,1.15) | 0.1151 | 0.50 (0.24,1.07) | 0.0742 |
| ≥8.0 | 1 |  | 1 |  | 1 |  | 1 |  |
| **Adjusted analysis** |  |  |  |  |  |  |  |  |
| <3.0 | 0.87 (0.62,1.21) | 0.3968 | 1.73 (0.40,7.48) | 0.4614 | 0.90 (0.47,1.74) | 0.7559 | 1.93 (1.09,3.42) | 0.0235 |
| 3.0–3.9 | 0.61 (0.43,0.87) | 0.0063 | 1.75 (0.34,9.08) | 0.5052 | 0.94 (0.47,1.86) | 0.8590 | 0.58 (0.27,1.26) | 0.1699 |
| 4.0–4.9 | 0.51 (0.37,0.69) | <0.0001 | 0.97 (0.19,4.96) | 0.9741 | 0.76 (0.41,1.38) | 0.3646 | 0.41 (0.21,0.82) | 0.0116 |
| 5.0–5.9 | 0.41 (0.30,0.55) | <0.0001 | 1.43 (0.33,6.10) | 0.6318 | 0.91 (0.51,1.60) | 0.7327 | 0.39 (0.20,0.75) | 0.0050 |
| 6.0–6.9 | 0.51 (0.38,0.69) | <0.0001 | 0.93 (0.18,4.69) | 0.9288 | 0.82 (0.45,1.49) | 0.5152 | 0.57 (0.30,1.08) | 0.0875 |
| 7.0–7.9 | 0.65 (0.47,0.89) | 0.0080 | 1.23 (0.24,6.20) | 0.8032 | 0.54 (0.26,1.15) | 0.1095 | 0.60 (0.29,1.22) | 0.1561 |
| ≥8.0 | 1 |  | 1 |  | 1 |  | 1 |  |

CI, confidence interval; HR, hazard ratio; IPTW, inverse probability of treatment weighting

^a^P-value was calculated by time-varying Cox proportional hazard analysis with periodic mean tacrolimus trough concentration as the time-varying exposure variable (unadjusted analysis). The adjusted analysis incorporated IPTW method with stabilized weight, which included: sex; age; previous dialysis months; use of immunosuppressants other than tacrolimus; use of induction agents; desensitization; donor age; donor sex; and serum creatinine (time-dependent covariate) for the calculation of stabilized weights.

**Table S9.** Crude incidence of allograft and patient outcomes 2–12 and 12–72 months post-transplant categorized by quartiles of tacrolimus trough level coefficient of variability

| **1-year outcomes** | |  |  | |  |  | | | |  |
| --- | --- | --- | --- | --- | --- | --- | --- | --- | --- | --- |
|  | Coefficient of variability | | | | | | | | | |
|  | <Q1  (<20.1%)  n=2,583 | | | Q1-Q2  (20.1-26.0%)  n=2,602 | | | Q2-Q3  (26.0-34.4%)  n=2,570 | ≥Q3  (≥34.4%)  n=2,528 | p-value^a^ | |
| Composite allograft outcome, n (%) | 147 (6.1) | | | 204 (8.5) | | | 312 (13.2) | 494 (21.5) | <0.0001 | |
| Biopsy-proven acute rejection | 114 (4.4) | | | 158 (6.1) | | | 257 (10.0) | 379 (15.0) | <0.0001 | |
| Kidney dysfunction (eGFR<30mL/min/1.73m^2^) | 17 (0.7) | | | 61 (2.3) | | | 119 (4.6) | 271 (10.7) | <0.0001 | |
| Development of de novo DSA | 38 (1.6) | | | 27 (1.1) | | | 25 (1.1) | 31 (1.3) | 0.3530 | |
| Death-censored graft failure | 5 (0.2) | | | 10 (0.4) | | | 22 (0.9) | 69 (2.7) | <0.0001 | |
| Severe infection, n (%) | 85 (3.4) | | | 146 (5.8) | | | 235 (9.5) | 377 (15.9) | <0.0001 | |
| Cardiovascular event, n (%) | 4 (0.2) | | | 3 (0.1) | | | 1 (0) | 7 (0.3) | 0.1583 | |
| Patient mortality, n (%) | 9 (0.3) | | | 6 (0.2) | | | 15 (0.6) | 48 (1.9) | <0.0001 | |
| **2–6-year outcomes** |  | | |  | | |  |  |  | |
|  | Coefficient of variability | | | | | | | | | |
|  | <Q1  (<24.2%)  n=1,135 | | | Q1-Q2  (24.2-30.4%)  n=1,145 | | | Q2-Q3  (30.4-39.4%)  n=1,115 | ≥Q3  (≥39.4%)  n=1,069 | p-value^a^ | |
| Composite allograft outcome, n (%) | 166 (15.2) | | | 213 (19.5) | | | 264 (25.1) | 387 (38.8) | <0.0001 | |
| Biopsy-proven acute rejection | 104 (9.2) | | | 137 (12.0) | | | 197 (17.7) | 291 (27.2) | <0.0001 | |
| Kidney dysfunction (eGFR<30mL/min/1.73m^2^) | 24 (2.1) | | | 63 (5.5) | | | 122 (10.9) | 225 (21.0) | <0.0001 | |
| Development of de novo DSA | 55 (5.1) | | | 71 (6.5) | | | 48 (4.6) | 63 (6.3) | 0.1485 | |
| Death-censored graft failure | 17 (1.5) | | | 25 (2.2) | | | 68 (6.1) | 155 (14.5) | <0.0001 | |
| Severe infection, n (%) | 73 (7.0) | | | 115 (10.9) | | | 158 (16.0) | 179 (19.8) | <0.0001 | |
| Cardiovascular event, n (%) | 2 (0.2) | | | 5 (0.4) | | | 10 (0.9) | 8 (0.8) | 0.0968 | |
| Malignancy, n (%) | 23 (2.1) | | | 34 (3.0) | | | 52 (4.7) | 48 (4.6) | 0.0013 | |
| Mortality, n (%) | 20 (1.8) | | | 14 (1.2) | | | 26 (2.3) | 67 (6.3) | <0.0001 | |

DSA, donor-specific antibodies; eGFR, estimated glomerular filtration; Q, quarter

^a^P-values from chi-square test.

**Table S10.** Multivariate Cox analysis of the risk of allograft and patient outcomes 2–12 and 12–72 months post-transplant across different tacrolimus trough level coefficient of variability groups

|  | Composite allograft outcome | | Biopsy-proven rejection | | Kidney dysfunction (eGFR<30mL/min/1.73m^2^) | | Development of de novo DSA | | Death-censored graft failure | |
| --- | --- | --- | --- | --- | --- | --- | --- | --- | --- | --- |
|  | aHR (95% CI) | p-value | aHR (95% CI) | p-value | aHR (95% CI) | p-value | aHR (95% CI) | p-value | aHR (95% CI) | p-value |
| **1-year outcomes** | | |  |  |  |  |  |  |  |  |
| ***CV – quartile groups*** | | |  |  |  |  |  |  |  |  |
| <Q1 | 0.46 (0.40,0.52) | <0.0001 | 0.29 (0.24,0.36) | <0.0001 | 0.07 (0.05,0.12) | <0.0001 | 0.94 (0.79,1.13) | 0.5080 | 0.11 (0.05,0.23) | <0.0001 |
| Q1–Q2 | 0.55 (0.49,0.62) | <0.0001 | 0.41 (0.34,0.49) | <0.0001 | 0.25 (0.19,0.33) | <0.0001 | 0.89 (0.75,1.07) | 0.2225 | 0.17 (0.10,0.31) | <0.0001 |
| Q2–Q3 | 0.73 (0.66,0.82) | <0.0001 | 0.68 (0.59,0.80) | <0.0001 | 0.48 (0.39,0.59) | <0.0001 | 0.94 (0.79,1.13) | 0.5147 | 0.31 (0.20,0.48) | <0.0001 |
| ≥Q3 | 1 |  | 1 |  | 1 |  | 1 |  | 1 |  |
| **2–6-year outcomes** | | |  |  |  |  |  |  |  |  |
| ***CV – quartile groups*** | | |  |  |  |  |  |  |  |  |
| <Q1 | 0.41 (0.35,0.47) | <0.0001 | 0.35 (0.29,0.43) | <0.0001 | 0.10 (0.07,0.15) | <0.0001 | 0.72 (0.56,0.92) | 0.0097 | 0.13 (0.09,0.20) | <0.0001 |
| Q1–Q2 | 0.49 (0.43,0.57) | <0.0001 | 0.45 (0.37,0.54) | <0.0001 | 0.24 (0.19,0.31) | <0.0001 | 0.87 (0.69,1.10) | 0.2517 | 0.17 (0.12,0.25) | <0.0001 |
| Q2–Q3 | 0.69 (0.61,0.79) | <0.0001 | 0.70 (0.59,0.82) | <0.0001 | 0.48 (0.39,0.59) | <0.0001 | 0.86 (0.67,1.09) | 0.2083 | 0.45 (0.35,0.58) | <0.0001 |
| ≥Q3 | 1 |  | 1 |  | 1 |  | 1 |  | 1 |  |

|  | Severe infection | | Cardiovascular  Event | | Patient mortality | | Malignancy | |
| --- | --- | --- | --- | --- | --- | --- | --- | --- |
|  | aHR (95% CI) | p-value | aHR (95% CI) | p-value | aHR (95% CI) | p-value | aHR (95% CI) | p-value |
| **1-year outcomes** | | |  |  |  |  |  |  |
| ***CV – quartile groups*** | | |  |  |  |  |  |  |
| <Q1 | 0.33 (0.28,0.40) | <0.0001 | 0.44 (0.17,1.14) | 0.0916 | 0.18 (0.09,0.37) | <0.0001 |  |  |
| Q1–Q2 | 0.48 (0.41,0.56) | <0.0001 | 0.82 (0.36,1.87) | 0.6411 | 0.12 (0.05,0.28) | <0.0001 |  |  |
| Q2–Q3 | 0.68 (0.59,0.78) | <0.0001 | 0.42 (0.16,1.11) | 0.0809 | 0.31 (0.17,0.53) | <0.0001 |  |  |
| ≥Q3 | 1 |  | 1 |  | 1 |  |  |  |
| **2–6-year outcomes** | | |  |  |  |  |  |  |
| ***CV – quartile groups*** | | |  |  |  |  |  |  |
| <Q1 | 0.50 (0.42,0.60) | <0.0001 | 0.87 (0.31,2.38) | 0.7793 | 0.31 (0.20,0.50) | <0.0001 | 0.47 (0.31,0.73) | 0.0008 |
| Q1–Q2 | 0.55 (0.46,0.65) | <0.0001 | 0.67 (0.24,1.85) | 0.4411 | 0.21 (0.12,0.36) | <0.0001 | 0.73 (0.50,1.07) | 0.1022 |
| Q2–Q3 | 0.80 (0.69,0.94) | 0.0051 | 1.22 (0.52,2.85) | 0.6451 | 0.42 (0.28,0.63) | <0.0001 | 0.86 (0.60,1.23) | 0.4186 |
| ≥Q3 | 1 |  | 1 |  | 1 |  | 1 |  |

aHR, adjusted hazard ratio; CI, confidence interval; CV, coefficient of variability; eGFR, estimated glomerular filtration

**Table S11.** Crude incidence of allograft and patient outcomes 2–12 and 12–72 months post-transplant categorized by tacrolimus time in therapeutic range groups

| **1 year outcomes** |  |  |  | | |
| --- | --- | --- | --- | --- | --- |
|  | High TTR (TTR ≥60%)  (n=1,012) | | | Low TTR (TTR<60%)  (n=9,271) | p-value |
| Composite allograft outcome, n (%) | 74 (8.1) | | | 1,083 (12.7) | 0.0001 |
| Biopsy-proven rejection | 83 (8.2) | | | 825 (8.9) | 0.4580 |
| Kidney dysfunction (eGFR<30mL/min/1.73m^2^) | 11 (1.1) | | | 457 (4.9) | <0.0001 |
| Development of de novo DSA | 4 (0.4) | | | 117 (1.4) | 0.0176 |
| Death-censored graft failure | 2 (0.2) | | | 104 (1.1) | 0.0057 |
| Severe infection, n (%) | 59 (6.0) | | | 784 (8.8) | 0.0025 |
| Cardiovascular event, n (%) | 0 | | | 15 (0.2) | 0.1979 |
| Patient mortality, n (%) | 6 (0.6) | | | 72 (0.8) | 0.5224 |
| **2–6 year outcomes** |  | | |  |  |
|  | High TTR (TTR ≥60%)  (n=1,035) | | | Low TTR (TTR<60%)  (n=3,429) | p-value |
| Composite allograft outcome, n (%) | 173 (17.5) | | | 857 (26.4) | <0.0001 |
| Biopsy-proven rejection | 129 (12.5) | | | 600 (17.5) | 0.0001 |
| Kidney dysfunction (eGFR<30mL/min/1.73m^2^) | 36 (3.5) | | | 398 (11.6) | <0.0001 |
| Development of de novo DSA | 51 (5.2) | | | 186 (5.7) | 0.4830 |
| Death-censored graft failure | 13 (1.3) | | | 252 (7.4) | <0.0001 |
| Severe infection, n (%) | 98 (10.6) | | | 427 (14.0) | 0.0089 |
| Cardiovascular event, n (%) | 5 (0.5) | | | 20 (0.6) | 0.6949 |
| Malignancy, n (%) | 34 (3.3) | | | 123 (3.6) | 0.6461 |
| Mortality, n (%) | 16 (1.5) | | | 111 (3.2) | 0.0041 |

DSA, donor-specific antibodies; eGFR, estimated glomerular filtration; TTR, time in therapeutic range

**Table S12.** Multivariate Cox analysis of the risk of allograft and patient outcomes 2–12 and 12–72 months post-transplant across high and low time in therapeutic range groups

| Tacrolimus trough level (ng/mL) | Composite  allograft outcome | | Biopsy-proven  acute rejection | | Kidney dysfunction (eGFR<30mL/min/1.73m^2^) | | Development of  de novo DSA | | Death-censored  graft failure | |
| --- | --- | --- | --- | --- | --- | --- | --- | --- | --- | --- |
|  | aHR (95% CI) | p-value | aHR (95% CI) | p-value | aHR (95% CI) | p-value | aHR (95% CI) | p-value | aHR (95% CI) | p-value |
| 1-year graft outcomes | | |  |  |  |  |  |  |  |  |
| High TTR (≥60%) | 0.95 (0.81,1.1) | 0.4909 | 0.91 (0.73,1.14) | 0.4263 | 0.23 (0.13,0.42) | <0.0001 | 1.27 (1.04,1.56) | 0.0204 | 0.25 (0.08,0.77) | 0.0162 |
| Low TTR | 1 |  | 1 |  | 1 |  | 1 |  | 1 |  |
| 2–6-year graft outcomes | | |  |  |  |  |  |  |  |  |
| High TTR (≥60%) | 0.66 (0.58,0.76) | <0.0001 | 0.69 (0.58,0.82) | <0.0001 | 0.31 (0.23,0.42) | <0.0001 | 0.87 (0.7,1.08) | 0.1917 | 0.25 (0.16,0.38) | <0.0001 |
| Low TTR | 1 |  | 1 |  | 1 |  | 1 |  | 1 |  |

|  | Severe infection | | Cardiovascular event | | Patient mortality | | Malignancy | |
| --- | --- | --- | --- | --- | --- | --- | --- | --- |
|  | aHR (95% CI) | p-value | aHR (95% CI) | p-value | aHR (95% CI) | p-value | aHR (95% CI) | p-value |
| 1-year patient outcomes | | |  |  |  |  |  |  |
| High TTR (≥60%) | 0.73 (0.58,0.9) | 0.0040 | –^a^ | 0.9890 | 0.75 (0.33,1.74) | 0.5071 |  |  |
| Low TTR | 1 |  | 1 |  | 1 |  |  |  |
| 2–6-year patient outcomes | | |  |  |  |  |  |  |
| High TTR (≥60%) | 0.85 (0.73,0.98) | 0.0264 | 1.14 (0.49,2.64) | 0.7554 | 0.58 (0.37,0.93) | 0.0230 | 0.86 (0.61,1.23) | 0.4154 |
| Low TTR | 1 |  | 1 |  | 1 |  | 1 |  |

aHR, adjusted hazard ratio; DSA, donor-specific antibodies; eGFR, estimated glomerular filtration; TTR, time in therapeutic range

^a^Hazard ratios for cardiovascular event were not estimable due to small number of outcome event.

**Figure S1. Data collection scheme illustrating the use of clinical data warehouses (CDW) from the five participating centers**


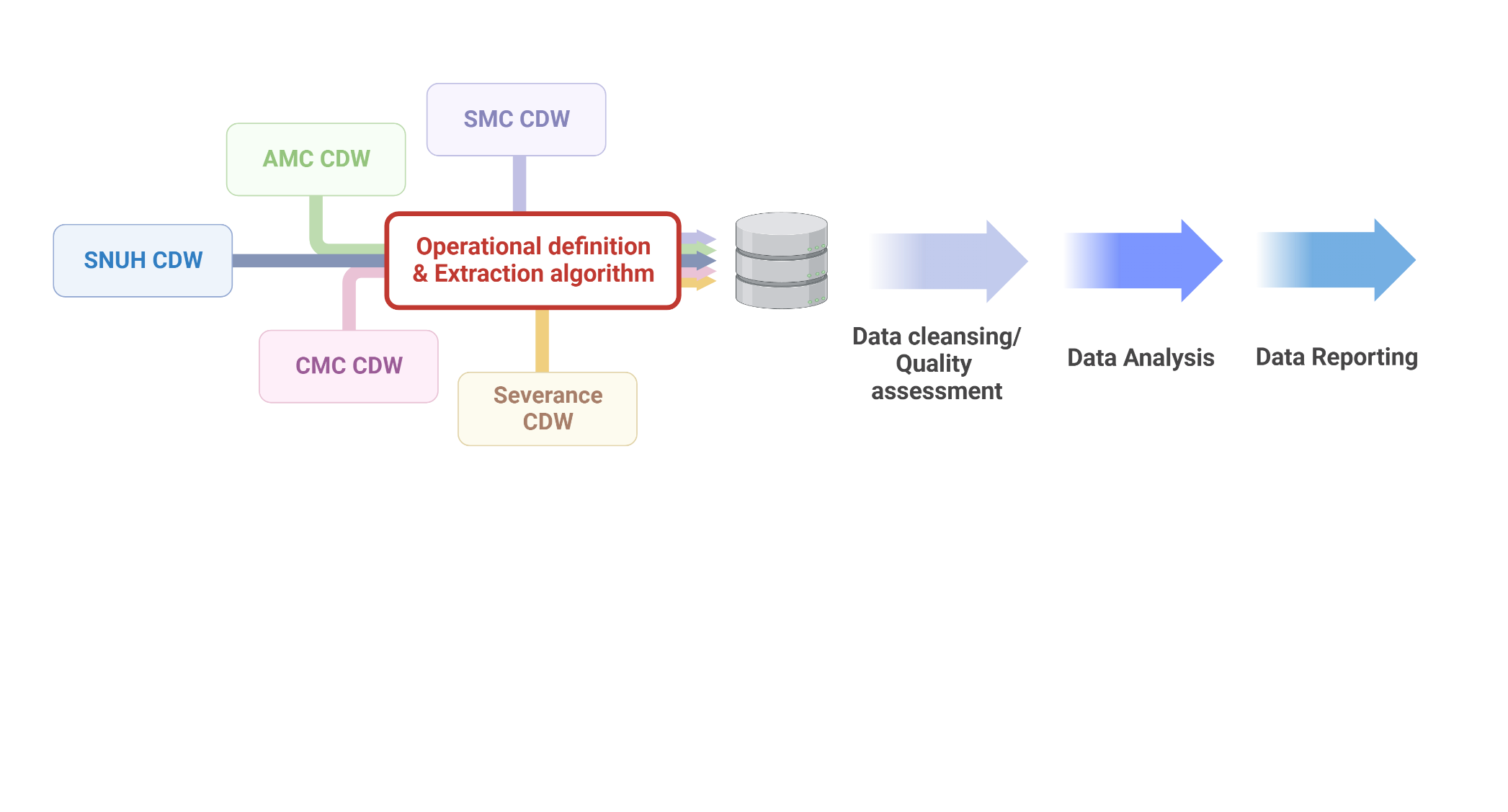

Supplement: SUPPLEMENTARY MATERIAL [file js9-110-6711-s001.docx]
